# Individuals redistribution based on differential evolution for covariance matrix adaptation evolution strategy

**DOI:** 10.1038/s41598-021-04549-1

**Published:** 2022-01-19

**Authors:** Zhe Chen, Yuanxing Liu

**Affiliations:** 1 China University of Geosciences, School of Computer Science, Wuhan, 430078 China; 2 Hubei Key Laboratory of Intelligent Geo-Information Processing, China University of Geosciences, Wuhan, 430078 China

**Keywords:** Computer science, Information technology

## Abstract

Among population-based metaheuristics, both Differential Evolution (DE) and Covariance Matrix Adaptation Evolution Strategy (CMA-ES) perform outstanding for real parameter single objective optimization. Compared with DE, CMA-ES stagnates much earlier in many occasions. In this paper, we propose CMA-ES with individuals redistribution based on DE, IR-CMA-ES, to address stagnation in CMA-ES. We execute experiments based on two benchmark test suites to compare our algorithm with nine peers. Experimental results show that our IR-CMA-ES is competitive in the field of real parameter single objective optimization.

## Introduction

The aim of real parameter single objective optimization is to find the best decision vector which can minimize (or maximize) an objective function in solution space. For years, real parameter single objective optimization is a hot spot of Artificial Intelligence (AI). A variety of population-based metaheuristics have been proposed in literature for the purpose.

Among types of population-based metaheuristic for real parameter single objective optimization, both Differential Evolution (DE)^1^ and Covariance Matrix Adaptation Evolution Strategy (CMA-ES)^2^ perform outstanding. In the six competitions on real parameter single objective optimization among population-based metaheuristics held by Congress of Evolutionary Computation (CEC), there are seven winners, including two joint winners in 2016. Among the seven winners, NBIPOP-aCMA-ES^3^ (2013) and HS-ES^4^ (2018) are based on CMA-ES, while L-SHADE^5^ (2014), L-SHADE-EpSin^6^ (2016), and IMODE? (2020) are based on DE. Moreover, UMOEAs-II^7^ (2016) is ensemble of CMA-ES and DE.

In execution of population-based metaheuristics, two phenomena, early convergence and stagnation, which both lead to the fact that no further improvement on solution can be made, are very common. The former phenomenon means that all individuals in population become same before a global optimum is found, while the other one means that difference between individuals is too low for operators of algorithm to obtain better solution although a global optimum is still not found. For non-trivial instance of real parameter single objective optimization, stagnation occurs much more often than early convergence in execution of types of population-based metaheuristics, including DE and CMA-ES.

The motivation of this paper is as below. Compared with DE, CMA-ES stagnates much earlier in many occasions. Therefore, measures, such as niching approach and restart strategy have been taken for years to help CMA-ES resist stagnation. Compared with niching approach, restart strategy makes more famous CMA-ES variants. For example, the winner in the CEC 2013 NBIPOP-aCMA-ES and the winner in the CEC 2018 HS-ES are both CMA-ES variants with restart. Beside restart, an improved version of univariate sampling is employed in HS-ES. The further comparison^8^ shows that HS-ES are one of the top performers among the six winners in the five competitions held in 2013, 2014, 2016, 2017, and 2018, respectively. It can be seen that, with the help of methods for resisting stagnation, CMA-ES performs better for real parameter single objective optimization than before. In fact, the above methods for resisting stagnation are very simple in idea. Now that such simple ideas are effective for improving CMA-ES, a more complicated strategy may be more promising. For example, DE may be a good choice for improving CMA-ES on resisting stagnation.

In fact, hybridisation techniques, such as memetic computing, are widely concerned in the field of AI^9^. Furthermore, there exist hybridisations of CMA-ES and another metaheuristic for different purposes. Examples are listed below. In CMA-ES/HDE^10^, CMA-ES and hybrid DE occupy a subpopulation, respectively. Migration occurs between the two subpopulations. In DCMA-EA^11,12^ operators of CMA-ES and those of DE are both used to produce new individuals. In UMOEAs-II^7^, CMA-ES and a variant of DE occupy a subpopulation, respectively. One of the constituent algorithms is executed in a part of generations, while both of them are executed in other generations. In above algorithms, CMA-ES works together with another constituent algorithm for search. A super-fit scheme based on CMA-ES is used to provide initialization for both re-sampled inheritance search and DE, respectively^13,14^. Here, CMA-ES is used as a method for initialization to replace random initialization.

In this paper, we propose CMA-ES with individuals redistribution based on DE, IR-CMA-ES. Once stagnation is detected, DE is executed to redistribute individuals. Here, stagnation is confirmed if improving ratio of the average fitness from the previous generation to the current one is lower than a threshold for a given number of successive generations. In a generation for DE, mutation and crossover of the original version of DE are executed and followed by offspring-surviving selection, which means that all offspring produced by mutation and crossover are selected, while parents are eliminated. Provided that CMA-ES is still caught in stagnation after a round of DE, a new round of DE with more generations is executed.

Compared with the existing hybridisations with CMA-ES, our algorithm is different in idea. Here, CMA-ES is used for search, while DE is used to redistribute individuals when CMA-ES faces towards stagnation. In our experiments based on the CEC 2014 and 2017 benchmark test suites, we compare our IR-CMA-ES with nine population-based metaheuristics. The experimental results show that our algorithm is competitive for real parameter single objective optimization.

## DE and CMA-ES for real parameter single objective optimization

In this section, the most popular methods for real parameter single objective optimization, CMA-ES and DE, are further introduced. Then, our idea is analyzed based on the features of CMA-ES and DE.

In population of DE, operators such as mutation, crossover, and selection, are exerted on individuals, i.e., target vectors. In the initial generation of population, target vectors $\vec x_{i,0} =(x_{1,i,0},x_{2,i,0},\ldots ,x_{D,i,0})$, where *i* is from 1 to *NP* and *NP* denotes the population size with dimensionality as *D* are produced randomly. In a given generation *g*, mutant vectors $\vec v_{i,g}$ are produced based on target vectors $\vec x_{i,g}$ by mutation. DE algorithms are compatible with different mutation strategies. Here, two mutation strategies among the popular ones, DE/rand/1 and DE/best/1, are presented in Eqs. (1) and (2) respectively, for instance

1 $$\begin{aligned}&\vec {v}_{i,g}=\vec {x}_{r1,g}+F\cdot (\vec {x}_{r2,g}-\vec {x}_{r3,g}), \end{aligned}$$

2 $$\begin{aligned}&\vec {v}_{i,g}=\vec {x}_{best,g}+F\cdot (\vec {x}_{r1,g}-\vec {x}_{r2,g}). \end{aligned}$$

In the equations, *r*1, *r*2 and *r*3 are distinct integers randomly chosen from the range [1, *NP*], and different from *i*. *F* is the scaling factor. $\vec {x}_{best,g}$ denotes the individual with the best fitness in the generation *g*. After mutation, trial vectors $\vec u_{i,g}=(u_{1,i,g}, u_{2,i,g},\ldots ,u_{D,i,g})$ are generated based on $\vec x_{i,g}$ and $\vec v_{i,g}$ by crossover. A widely used crossover strategy—binomial crossover—is

3 $$\begin{aligned} u_{j,i,g}= \left\{ \begin{array}{lr} v_{j,i,g}, &{} if~rand(0,1)\le Cr~or~j=randn(i),\\ x_{j,i,g}, &{} otherwise, \end{array} \right. \end{aligned}$$

where $Cr\in [0,1]$ is the crossover rate, and *randn*(*i*) is an integer randomly generated from the range [1, *NP*] to ensure that $\vec {u}_{i,g}$ has at least one component from $\vec {v}_{i,g}$. In DE, crossover and mutation together are specified as trial vector generation strategy. For selection, the operation is

4 $$\begin{aligned} \vec {x}_{i,g+1}= \left\{ \begin{array}{lr} \vec {u}_{i,g}, &{} if~f(\vec {u}_{i,g})\le f(\vec {x}_{i,g}),\\ \vec {x}_{i,g}, &{} otherwise, \end{array} \right. \end{aligned}$$

where $f(\vec {u}_{i,g})$ and $f(\vec {x}_{i,g})$ represent fitness of $\vec {u}_{i,g}$ and $\vec {x}_{i,g}$, respectively.

In population of CMA-ES, the $g+1$th generation is obtained based on the *g*th generation as follow,

5 $$\begin{aligned} \varvec{x}_{k}^{(g+1)}=\langle \varvec{x}\rangle _{\mu }^{(g)}+\sigma ^{(g)} \underbrace{\varvec{B}^{(g)} \varvec{D}^{(g)} \varvec{z}_{k}^{(g+1)}}_{\sim \mathcal {N}\left( \mathbf {0}, \varvec{C}^{(g)}\right) } \quad , \quad k=1, \ldots , \lambda , \end{aligned}$$

where

6 $$\begin{aligned} \langle \varvec{x}\rangle _{\mu }^{(g)}=\frac{1}{\mu } \sum _{i \in I_{s e l}^{(g)}} \varvec{x}_{i}^{(g)} \end{aligned}$$

represents the center of mass of the selected individuals in the *g*th generation, while $I_{s e l}^{(g)}$ is the set of indices of the same individuals with $\left| I_{s e l}^{(g)}\right| =\mu \cdot \sigma ^{(g)}$ is the global step size. The random vectors $z_{k}$ in Eq. (5) are $\mathcal {N}(\mathbf {0}, \varvec{I})$ distributed (*n*-dimensional normally distributed with expectation zero and the identity covariance matrix) and serve to generate offspring. We can calculate their center of mass as

7 $$\begin{aligned} {\langle z\rangle }_{\mu }^{(g+1)}=\frac{1}{\mu } \sum _{i \in I_{s e l}^{(g+1)}} \varvec{z}_{i}^{(g+1)} \end{aligned}$$

The covariance matrix $\varvec{C}^{(g)}$ of the random vectors $\varvec{B}^{(g)} \varvec{D}^{(g)} \varvec{z}_{k}^{(g+1)}$ is a symmetrical positive $n \times n$-matrix. The columns of the orthogonal matrix $\varvec{B}^{(g)}$ represent normalized eigenvectors of the covariance matrix. $\varvec{D}^{(g)}$ is a diagonal matrix whose elements are the square roots of the eigenvalues of $\varvec{C}^{(g)}$. Hence, the relation of $\varvec{B}^{(g)}$ and $\varvec{D}^{(g)}$ to $\varvec{C}^{(g)}$ can be expressed by

8 $$\begin{aligned} \varvec{C}^{(g)}=\varvec{B}^{(g)} \varvec{D}^{(g)}\left( \varvec{B}^{(g)} \varvec{D}^{(g)}\right) ^{\mathrm {T}} \quad \text{ and } \quad \varvec{C}^{(g)} \varvec{b}_{i}^{(g)}=\left( d_{i i}^{(g)}\right) ^{2} \cdot \varvec{b}_{i}^{(g)} \end{aligned}$$

where $\varvec{b}_{i}^{(g)}$ represents the *i*-th column of $\varvec{B}^{(g)}$ and $\left\| \varvec{b}_{i}^{(g)}\right\| =1$ and $d_{i i}^{(g)}$ are the diagonal elements of $\varvec{D}^{(g)}$. Surfaces of equal probability density of the random vectors $\varvec{B}^{(g)} \varvec{D}^{(g)} \varvec{z}_{k}^{(g+1)} \sim \mathcal {N}\left( \mathbf {0}, \varvec{C}^{(g)}\right)$ are (hyper-)ellipsoids whose main axes correspond to the eigenvectors of the covariance matrix. The squared lengths of the axes are equal to the eigenvalues of the covariance matrix.

Either CMA-ES or DE is based on population. It can be seen that DE is much simpler in steps than CMA-ES. Hence, to resist stagnation, modification based on DE is easier than that based on CMA-ES. According to our idea, when CMA-ES is trapped in stagnation, DE takes over population. As a result, individuals are produced in a new manner. If the new individuals survive selection, the state of stagnation may be broken since distribution of population changes significantly. Details of our method are shown in “Methods”.

## Results and discussion

In our experiments, our IR-CMA-ES is compared with nine population-based metaheuristics, L-SHADE^5^, UMOEAs-II^7^, jSO^15^, L-PalmDE^16^, HS-ES^4^, HARDDE^17^, NDE^18^, PaDE^19^, and CSDE^20^. Among the above competitors selected by us, UMOEAs-II is based on both CMA-ES and DE, while HS-ES is based on CMA-ES. The other algorithms are all DE variants. After all, compared with CMA-ES, DE has much more recent variants. Our experiments are based on the CEC 2014 and 2017 benchmark testing suites. Settings of the peers from literature are shown in Table 1, where *D* denotes dimensionality.

**Table 1:** Settings of the involved peers.

Algorithm	Parameters
L-SHADE	$NP_{max}=D\cdot 18$, $NP_{min}=4$, $|A|=NP_{max}\cdot 2.6$, $H=6$, and $p=0.11$^5^
UMOEAs-II	$NP_{max}=D\cdot 18+4+\lfloor log(D)\cdot 3 \rfloor$, $NP_{min}=8+\lfloor log(D)\cdot 3 \rfloor$ , $prob_{ls}=0.1$, and $cfe_{LS}=D\cdot 2000$^7^
jSO	$NP_{max}=25\cdot log(D)\cdot \sqrt{D}$, $NP_{min}=4$ $p_{max}=0.25$, $p_{min}=\frac{p_{max}}{2}$, $|A|=1404$, H=5
L-PalmDE	$NP_{max}=D\cdot 23$, $NP_{min}=k$, $k=8$, $p=0.1$, $a=1.6$, and $T_0=70$
HS-ES	$NP_1=200$, $NP_2=80+\lfloor ln(D)\cdot 3 \rfloor$, $NP_3=450$ when $D=50$, $NP_3=600$ when $D=100$, $cc=0.96$, $I=20$,
	$N^{Step 1}=100$, $N^{Step 4}=360$ when $D=50$, and $N^{Step 4}=480$ when $D=100$^4^
HARDDE	$NP=log(D)\cdot \sqrt{D}\cdot 25\sim 4$, $H=4$, $p=0.11$, and $r^{arc}=3$
NDE	$NP_{max}=300$, $NP_{min}=5$, $gm=10$, and $c=0.1$^18^
PaDE	$NP=log(D)\cdot \sqrt{D}\cdot 25\sim 4$, $k=4$, $p=0.11$, $r^{arc}=1.6$, $T_0=70$, and $r^d=0.04$
CSDE	$NP=log(D)\cdot \sqrt{D}\cdot 25\sim K$, $p=0.25\sim 0.05$, $K=6$, $r^{rac,A}=1.6$, $r^{rac,B}=5$, $T_0=\frac{G_max}{2}$, $N=D\cdot 2$, $\xi =0.01$

The criterion for termination, maximum number of fitness evaluations, is set 10,000 $\cdot D$ in experiment.

### Results for the CEC 2014 benchmark testing suite

**Figure 1:**
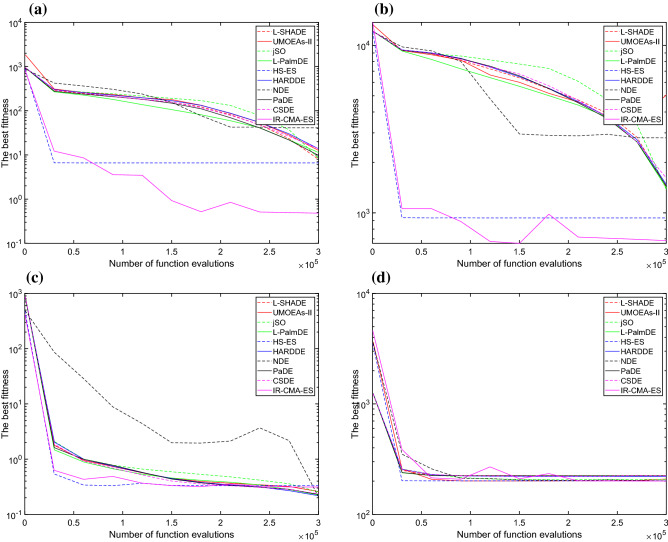
Convergence graphs of the six algorithms for the for functions from the CEC 2014 benchmark test suite. **a** F9, **b** F11, **c** F14, and **d** F24.

When $D=30$, 50, and 100, the peers and our algorithm are executed 30 times for each function, respectively. The results are given in Tables 2, 3 and 4. We notice that, when dimensionality is set 30, for F9, F11, F14, and F24 in the CEC 2014 suite, our algorithm loses to no peer. Thus, we give the convergence graph of all the algorithms for the functions with 30 in dimensionality in Fig. 1.

**Table 2:** Results of the ten algorithms for the CEC 2014 functions with 30 in dimensionality.

Function	Average (standard deviation)									
	L-SHADE	UMOEAs-II	jSO	L-PalmDE	HS-ES	HARDDE	NDE	PaDE	CSDE	IR-CMA-ES
F1	9.47E−15	1.96E−11	0.00E+00	1.37E−14	3.57E−10	6.39E−14	5.50E+02	1.18E−14	9.47E−15	8.14E−10
	(7.77E−15)$\approx$	(3.25E−11)$\approx$	(0.00E+00)$\approx$	(5.88E−15)$\approx$	(3.20E−10)$\approx$	(5.68E−14)$\approx$	(1.31E+03)−	(5.39E−15)$\approx$	(6.81E−15)$\approx$	(1.34E−09)
F2	0.00E+00	1.14E−14	0.00E+00	9.47E−16	3.27E−10	3.79E−15	0.00E+00	0.00E+00	0.00E+00	0.00E+00
	(0.00E+00)$\approx$	(1.42E−14)$\approx$	(0.00E+00)$\approx$	(5.19E−15)$\approx$	(6.50E−10)−	(9.83E−15)$\approx$	(0.00E+00)$\approx$	(0.00E+00)$\approx$	(0.00E+00)$\approx$	(0.00E+00)
F3	0.00E+00	7.58E−15	0.00E+00	3.79E−15	3.10E−10	0.00E+00	0.00E+00	0.00E+00	0.00E+00	1.26E−14
	(0.00E+00)$\approx$	(1.97E−14)$\approx$	(0.00E+00)$\approx$	(2.08E−14)$\approx$	(4.60E−10)$\approx$	(0.00E+00)$\approx$	(0.00E+00)$\approx$	(0.00E+00)$\approx$	(0.00E+00)$\approx$	(4.56E−09)
F4	5.49E−14	4.36E−14	0.00E+00	3.98E−14	3.05E−11	5.87E−14	7.74E−04	5.12E−14	3.03E−14	5.79E−14
	(2.79E−14)$\approx$	(3.23E−14)$\approx$	(0.00E+00)$\approx$	(2.65E−14)$\approx$	(4.41E−11)$\approx$	(2.79E−14)$\approx$	(1.53E−03)−	(2.29E−14)$\approx$	(2.88E−14)$\approx$	(5.46E−10)
F5	2.01E+01	2.00E+01	2.08E+01	2.01E+01	2.00E+01	2.02E+01	2.02E+01	2.02E+01	2.01E+01	2.00E+01
	(2.71E−02)−	(7.12E−04)$\approx$	(2.42E−01)−	(7.19E−02)−	(5.65E−04)$\approx$	(4.45E−02)−	(8.50E−02)−	(3.96E−02)−	(1.78E−02)−	(3.57E−03)
F6	0.00E+00	2.29E−05	8.52E−06	7.46E−01	7.89E−01	2.35E−05	2.38E+00	0.00E+00	7.30E−05	3.57E−03
	(0.00E+00)$+$	(9.53E−05)$+$	(1.26E−05)$+$	(2.35E+00)−	(1.23E+00)−	(5.83E−05)$+$	(2.29E+00)−	(0.00E+00)$+$	(3.75E−04)$+$	(7.45E−01)
F7	0.00E+00	3.79E−15	0.00E+00	0.00E+00	1.97E−11	0.00E+00	0.00E+00	0.00E+00	0.00E+00	0.00E+00
	(0.00E+00)$\approx$	(2.08E−14)$\approx$	(0.00E+00)$\approx$	(0.00E+00)$\approx$	(6.25E−11)$\approx$	(0.00E+00)$\approx$	(0.00E+00)$\approx$	(0.00E+00)$\approx$	(0.00E+00)$\approx$	(0.00E+00)
F8	1.40E−13	9.10E−14	0.00E+00	6.44E−14	8.69E+00	3.26E−13	2.32E+00	1.25E−13	4.55E−14	3.59E−01
	(6.46E−14)$+$	(4.63E−14)$+$	(0.00E+00)$+$	(5.73E−14)$+$	(2.63E+00)−	(7.15E−14)$+$	(2.93E+00)−	(4.58E−14)$+$	(7.67E−14)$+$	(8.35E−01)
F9	6.77E+00	7.63E−01	8.38E+00	1.20E+01	7.06E+00	1.19E+01	4.50E+01	8.34E+00	1.11E+01	3.75E−01
	(1.56E+00)−	(8.54E−01)−	(1.87E+00)−	(2.76E+00)−	(2.62E+00)−	(1.91E+00)−	(2.15E+01)−	(2.00E+00)−	(1.59E+00)−	(1.18E+00)
F10	2.78E−03	2.08E−03	1.63E+00	1.46E−12	4.17E+02	3.03E−12	4.37E+00	2.08E−03	1.39E−03	5.87E+00
	(7.20E−03)$+$	(8.38E−03)$+$	(1.17E+00)$+$	(1.21E−12)$+$	(2.33E+02)−	(9.94E13)$+$	(2.27E+01)$\approx$	(6.35E−03)$+$	(5.28E−03)$+$	(5.64E+00)
F11	1.22E+03	1.22E+03	1.31E+03	1.40E+03	6.85E+02	1.23E+03	2.10E+03	1.25E+03	1.23E+03	5.97E+02
	(1.75E+02)−	(2.35E+02)−	(2.29E+02)−	(3.40E+02)−	(3.33E+02)$\approx$	(1.99E+02)−	(5.59E+02)−	(2.04E+02)−	(2.51E+02)−	(7.48E+02)
F12	1.62E−01	9.38E−02	4.27E−01	1.48E−01	1.69E−02	1.82E−01	1.55E−01	1.82E−01	1.39E−01	1.46E−02
	(2.62E−02)−	(5.50E−02)−	(3.53E−01)−	(7.04E−02)$\approx$	(1.51E−02)$\approx$	(4.50E−02)−	(1.02E−01)−	(3.56E−02)−	(2.14E−02)−	(1.73E−02)
F13	1.19E−01	1.03E−01	1.35E−01	1.03E−01	4.91E−02	1.48E−01	9.90E−02	1.14E−01	1.39E−01	9.44E−02
	(1.83E−02)$\approx$	(2.67E−02)$\approx$	(2.13E−02)−	(2.22E−02)$\approx$	(1.17E−02)$+$	(2.71E−02)−	(3.03E−02)$\approx$	(1.36E−02)$\approx$	(2.38E−02)−	(4.38E−02)
F14	2.40E−01	2.34E−01	2.24E−01	2.33E−01	3.31E−01	2.13E−01	2.26E−01	2.10E−01	2.10E−01	1.86E−01
	(2.90E−02)−	(2.41E−02)−	(3.46E−02)−	(2.80E−02)−	(4.97E−02)−	(2.90E−02)−	(3.66E−02)−	(2.50E−02)−	(2.60E−02)−	(4.53E−02)
F15	2.17E+00	2.02E+00	2.22E+00	2.07E+00	3.01E+00	2.37E+00	3.10E+00	2.20E+00	2.22E+00	2.84E+00
	(2.04E−01)$+$	(3.78E−01)$+$	(3.21E−01)$+$	(3.04E−01)$+$	(6.55E−01)$\approx$	(3.06E−01)−	(8.71E−01)−	(2.22E−01)$+$	(2.66E−01)$+$	(7.73E−01)
F16	8.56E+00	9.02E+00	8.72E+00	8.77E+00	1.01E+01	8.87E+00	1.00E+01	8.48E+00	8.85E+00	1.12E+01
	(2.95E−01)$+$	(6.42E−01)$+$	(7.48E−01)$\approx$	(7.91E−01)$+$	(9.20E−01)$+$	(3.98E−01)$+$	(7.255E−01)$+$	(4.81E−01)$+$	(3.81E−01)$+$	(6.62E−01)
F17	2.09E+02	1.84E+02	6.74E+01	2.07E+02	2.13E+01	1.06E+02	1.91E+02	1.94E+02	1.01E+02	2.24E+01
	(1.07E+02)−	(9.06E+01)−	(1.52E+01)−	(9.57E+01)−	(3.92E+01)$\approx$	(5.86E+01)−	(1.19E+02)−	(1.06E+02)−	(5.78E+01)−	(3.13E+01)
F18	6.14E+00	5.04E+00	2.21E+00	6.08E+00	5.72E+00	3.62E+00	8.58E+00	6.43E+00	5.24E+00	4.12E+00
	(2.83E+00)$\approx$	(2.49E+00)−	(1.18E+00)$+$	(2.47E+00)−	(2.76E+00)−	(1.51E+00)$+$	(3.77E+00)−	(2.94E+00)$\approx$	(2.75E+00)$\approx$	(4.56E+00)
F19	3.64E+00	3.03E+00	1.99E+00	3.00E+00	2.94E+00	2.81E+00	2.82E+00	3.43E+00	2.88E+00	2.13E+00
	(5.19E−01)−	(7.93E−01)−	(6.54E−01)$\approx$	(5.01E−01)−	(8.01E−01)−	(4.04E−01)−	(7.11E−01)−	(5.75E−01)−	(5.84E−01)−	(8.44E−01)
F20	2.87E+00	3.59E+00	2.02E+00	3.55E+00	3.65E+00	3.15E+00	5.30E+00	2.60E+00	2.99E+00	4.52E+00
	(1.15E+00)$+$	(1.26E+00)$+$	(6.92E−01)$+$	(1.32E+00)$+$	(8.23E+00)$+$	(1.34E+00)$+$	(1.70E+00)$\approx$	(1.39E+00)$+$	(9.56E−01)$+$	(7.96E+00)
F21	1.04E+02	5.47E+01	2.62E+01	9.59E+01	2.07E+01	5.91E+01	4.06E+01	8.82E+01	4.10E+01	2.64E+01
	(7.62E+01)−	(6.40E+01)−	(3.90E+01)$\approx$	(7.85E+01)−	(6.06E+01)$\approx$	(8.24E+01)−	(5.14E+01)−	(6.82E+01)−	(5.61E+01)−	(3.34E+01)
F22	2.38E+01	2.76E+01	2.91E+01	4.96E+01	1.636E+02	8.06E+01	6.40E+01	7.18E+01	8.50E+01	1.98E+02
	(1.44E+00)$+$	(6.16E+00)$+$	(2.18E+01)$+$	(4.80E+01)$+$	(7.39E+01)$+$	(5.49E+01)$+$	(6.83E+01)$+$	(5.59E+01)$+$	(5.59E+01)$+$	(9.48E+01)
F23	3.15E+02	2.00E+02	3.15E+02	3.15E+02	3.15E+02	3.15E+02	3.15E+02	3.15E+02	3.15E+02	3.15E+02
	(5.78E−14)$\approx$	(0.00E+00)$+$	(1.09E−13)$\approx$	(5.78E−14)$\approx$	(7.71E−06)$\approx$	(5.78E−14)$\approx$	(1.46E−13)$\approx$	(5.78E−14)$\approx$	(5.78E−14)$\approx$	(4.33E−05)
F24	2.25E+02	2.00E+02	2.07E+02	2.24E+02	2.24E+02	2.22E+02	2.21E+02	2.23E+02	2.22E+02	2.00E+02
	(2.33E+00)−	(8.44E−14)$\approx$	(1.02E+01)−	(1.01E+00)−	(2.32E+00)−	(4.25E+00)−	(6.99E+00)−	(9.73E−01)−	(1.02E+00)−	(7.39E−13)
F25	2.03E+02	2.00E+02	2.03E+02	2.03E+02	2.09E+02	2.03E+02	2.03E+02	2.03E+02	2.03E+02	2.00E+02
	(5.29E−02)−	(0.00E+00)$\approx$	(2.80E−02)−	(1.04E−01)−	(1.92E+00)−	(4.69E−02)−	(2.88E−01)−	(1.30E−01)−	(4.68E−02)−	(0.00E+00)
F26	1.00E+02	1.00E+02	1.00E+02	1.00E+02	1.37E+02	1.00E+02	1.00E+02	1.00E+02	1.00E+02	1.00E+02
	(1.40E−02)$\approx$	(2.50E−02)$\approx$	(2.22E−02)$\approx$	(1.46E−13)$\approx$	(4.32E+01)−	(2.65E−02)$\approx$	(5.82E−02)$\approx$	(1.34E−02)$\approx$	(1.56E−02)$\approx$	(3.52E+00)
F27	3.00E+02	2.00E+02	3.00E+02	3.00E+02	3.02E+02	3.00E+02	4.00E+02	3.00E+02	3.00E+02	3.60E+02
	(8.44E−14)$+$	(0.00E+00)$+$	(1.71E−13)$+$	(1.46E−13)−	(1.16E+01)$+$	(2.85E−13)$+$	(2.40E−01)−	(1.46E−13)$+$	(0.00E+00)$+$	(2.75E+01)
F28	8.32E+02	2.00E+02	8.25E+02	8.57E+02	8.97E+02	8.51E+02	8.26E+02	8.54E+02	8.45E+02	7.87E+02
	(1.95E+01)$\approx$	(0.00E+00)$+$	(1.94E+01)$\approx$	(1.91E+01)$\approx$	(2.503E+01)−	(1.66E+01)$\approx$	(2.68E+01)−	(1.90E+01)$\approx$	(1.80E+01)$\approx$	(5.05E+01)
F29	7.17E+02	7.16E+02	7.16E+02	6.80E+02	2.58E+02	5.31E+02	6.47E+02	7.00E+02	6.25E+02	2.59E+02
	(4.47E+00)−	(2.56E+00)−	(2.48E+00)−	(1.39E+02)−	(7.81E+01)$\approx$	(2.67E+02)−	(1.84E+02)−	(9.32E+01)−	(2.11E+02)−	(1.75E+01)
F30	1.29E+03	9.26E+02	6.53E+02	5.84E+02	1.75E+03	4.79E+02	5.62E+02	5.88E+02	4.57E+02	5.35E+02
	(5.41E+02)−	(3.51E+02)−	(2.19E+02)$\approx$	(2.02E+02)$\approx$	(3.984E+02)−	(1.30E+02)$\approx$	(1.86E+02)$\approx$	(2.42E+02)$\approx$	(5.30E+01)$\approx$	(5.66E+02)
−	12	10	10	13	13	13	19	11	12	
$+$	8	10	8	6	5	8	2	8	8	
$\approx$	10	10	12	11	12	9	9	11	10	

“$+$” or “−” denotes that the current result is significantly better or statistical worse than the result of our IR-CMA-ES in terms of Wilcoxon’s rank sum test at a 0.05 significance level, respectively. Meanwhile, “$\approx$” represents that there is no significant difference.

**Table 3:** Results of the ten algorithms for the CEC 2014 functions with 50 in dimensionality.

Function	Average (standard deviation)									
	L-SHADE	UMOEAs-II	jSO	L-PalmDE	HS-ES	HARDDE	NDE	PaDE	CSDE	IR-CMA-ES
F1	6.05E+02	1.79E−03	9.04E+00	2.76E+03	2.26E−09	6.18E+01	1.07E+05	1.03E+03	2.34E+02	5.49E−08
	(1.13E+03)−	(1.05E−03)−	(1.42E+01)−	(2.88E+03)−	(3.47E−09)$\approx$	(2.26E+02)−	(4.91E+04)−	(2.41E+03)−	(6.96E+02)−	(6.47E−12)
F2	3.51E−14	2.62E−13	0.00E+00	3.88E−14	1.87E−09	6.92E−14	3.84E−03	3.88E−14	2.94E−14	5.78E−14
	(1.22E−14)$\approx$	(1.94E−13))$\approx$	(0.00E+00)$\approx$	(1.39E−14)$\approx$	(2.33E−09)−	(2.07E−14)$\approx$	(7.73E−03)−	(1.39E−14)$\approx$	(5.19E−15)$\approx$	(7.17E−14)
F3	5.68E−14	1.14E−13	0.00E+00	5.68E−14	1.20E−09	5.68E−14	5.50E−05	5.31E−14	4.17E−14	7.48E−10
	(0.00E+00)+	(3.66E−14)+	(0.00E+00)+	(0.00E+00)+	(1.75E−09)$\approx$	(0.00E+00)+	(7.70E−05)−	(1.44E−14)+	(2.56E−14)+	(6.44E−10)
F4	3.41E+01	3.93E+01	5.58E+01	1.01E+01	3.25E+00	1.02E+01	5.53E+01	1.67E+01	4.60E−01	2.35E+00
	(4.61E+01)−	(4.89E+01)−	(4.84E+01)−	(2.98E+01)−	(1.78E+01)$\approx$	(2.98E+01)−	(4.17E+01)−	(3.70E+01)−	(3.42E−01)+	(2.48E+00)
F5	2.03E+01	2.00E+01	2.08E+01	2.02E+01	2.00E+01	2.03E+01	2.03E+01	2.03E+01	2.01E+01	2.00E+01
	(2.50E−02)−	(2.20E−04))$\approx$	(3.49E−01)−	(1.58E−01)−	(1.16E−04)$\approx$	(8.05E−02)−	(1.21E−01)−	(4.64E−02)−	(3.65E−02)−	(2.64E−04)
F6	3.47E−01	1.61E−01	5.73E−02	7.82E−01	4.07E−05	2.12E−05	9.80E+00	1.46E−01	1.85E−03	7.58E−04
	(6.27E−01)−	(3.20E−01)−	(2.82E−01)−	(3.49E+00)−	(8.37E−05)+	(3.44E−05)+	(4.10E+00)−	(3.81E−01)−	(5.84E−03)−	(3.27E−05)
F7	9.47E−14	1.14E−13	0.00E+00	1.02E−13	9.67E−10	9.85E−14	1.07E+00	8.34E−14	3.79E−15	7.97E−10
	(4.31E−14)+	(0.00E+00)+	(0.00E+00)+	(3.47E−14)+	(2.19E−09)$\approx$	(3.93E−14)+	(2.80E−03)−	(5.11E−14)+	(2.08E−14)+	(0.00E+00)
F8	1.13E−10	2.11E−12	0.00E+00	1.48E−13	1.53E+00	6.56E−13	8.82E+00	7.58E−13	1.52E−13	1.75E+00
	(1.16E−10)+	(1.11E−12)+	(0.00E+00)+	(6.08E−14)+	(1.27E+00)$\approx$	(1.57E−13)+	(5.19E+00)−	(1.56E−13)+	(1.09E−13)+	(2.64E+00)
F9	1.14E+01	4.15E+00	1.49E+01	2.16E+01	8.29E−01	2.45E+01	5.85E+01	1.64E+01	2.06E+01	6.01E−01
	(2.43E+00)−	(1.46E+00)−	(2.95E+00)−	(4.19E+00)−	(7.43E−01)$\approx$	(3.38E+00)−	(3.19E+01)−	(2.44E+00)−	(2.87E+00)−	(9.46E−01)
F10	4.28E−02	5.36E−01	1.05E+01	1.08E−02	2.04E+02	4.46E−03	1.21E+01	7.96E−03	4.24E−03	5.08E+01
	(2.43E−02)+	(5.69E−01)+	(3.03E+00)+	(1.79E−02)+	(1.59E+02)−	(7.38E−03)+	(6.58E+01)+	(9.01E−03)+	(6.79E−03)+	(7.85E+01)
F11	3.33E+03	3.50E+03	3.27E+03	3.66E+03	5.55E+02	3.28E+03	4.95E+03	3.27E+03	3.35E+03	3.94E+02
	(3.12E+02)−	(5.84E+02)−	(3.61E+02)−	(5.08E+02)−	(2.35E+02)−	(2.89E+02)−	(8.09E+02)−	(2.95E+02)−	(2.56E+02)−	(6.37E+02)
F12	2.19E−01	1.23E−01	2.57E−01	2.19E−01	2.48E−02	2.12E−01	1.46E−01	2.25E−01	1.72E−01	1.51E−02
	(2.31E−02)−	(7.33E−02)−	(3.94E−02)−	(7.95E−02)−	(2.38E−02)−	(5.20E−02)−	(1.10E−01)−	(2.82E−02)−	(2.40E−02)−	(3.10E−02)
F13	1.65E−01	1.50E−01	2.02E−01	1.66E−01	7.02E−02	2.18E−01	1.60E+00	1.86E−01	2.03E−01	1.03E−01
	(1.74E−02)−	(3.26E−02)−	(2.74E−02)−	(3.13E−02)−	(1.19E−02)+	(2.64E−02)−	(4.19E−02)−	(2.11E−02)−	(1.59E−02)−	(1.26E−02)
F14	3.07E−01	2.91E−01	2.90E−01	3.04E−01	3.99E−01	2.75E−01	2.83E−01	2.94E−01	2.82E−01	2.83E−01
	(2.16E−02)$\approx$	(2.74E−02))$\approx$	(4.75E−02)$\approx$	(3.09E−02)$\approx$	(4.13E−02)−	(2.12E−02)$\approx$	(4.22E−02)$\approx$	(2.26E−02)$\approx$	(2.43E−02)$\approx$	(3.70E−02)
F15	5.11E+00	5.27E+00	5.31E+00	4.42E+00	4.74E+00	5.27E+00	5.49E+00	5.06E+00	5.16E+00	5.00E+00
	(4.56E−01)$\approx$	(9.35E−01)−	(6.43E−01)−	(7.77E−01)+	(9.54E−01)+	(6.24E−01)−	(1.04E+00)−	(5.45E−01)$\approx$	(4.56E−01)−	(9.92E−01)
F16	1.68E+01	1.80E+01	1.70E+01	1.71E+01	1.86E+01	1.71E+01	1.89E+01	1.68E+01	1.72E+01	1.87E+01
	(4.18E−01)+	(1.03E+00)$\approx$	(7.79E−01)+	(8.90E−01)+	(8.73E−01)$\approx$	(4.46E−01)+	(6.35E−01)$\approx$	(3.71E−01)+	(4.00E−01)+	(4.29E−01)
F17	1.59E+03	1.11E+03	3.48E+02	1.71E+03	1.60E+03	7.81E+02	9.10E+02	1.69E+03	9.75E+02	1.09E+03
	(4.34E+02)−	(3.85E+02))$\approx$	(1.94E+02)+	(3.50E+02)−	(1.33E+03)−	(2.56E+02)+	(3.77E+02)+	(3.92E+02)−	(2.51E+02)$\approx$	(7.65E+02)
F18	1.01E+02	7.30E+01	1.18E+01	1.05E+02	8.30E−01	3.62E+01	3.14E+01	1.05E+02	8.05E+01	8.47E−01
	(1.56E+01)−	(1.92E+01)−	(4.68E+00)−	(1.33E+01)−	(6.57E−01)$\approx$	(1.43E+01)−	(1.06E+01)−	(1.45E+01)−	(1.04E+01)−	(5.95E−01)
F19	8.20E+00	8.33E+00	9.47E+00	8.49E+00	7.15E+00	8.81E+00	1.01E+01	7.91E+00	8.19E+00	5.74E+00
	(1.93E+00)−	(2.19E+00)−	(7.54E−01)−	(1.60E+00)−	(9.26E−01)−	(1.92E+00)−	(6.84E−01)−	(1.74E+00)−	(1.80E+00)−	(6.63E−01)
F20	1.35E+01	1.40E+01	5.79E+00	1.58E+01	2.27E+00	1.13E+01	3.22E+01	1.35E+01	1.10E+01	7.49E+00
	(4.78E+00)−	(4.80E+00)−	(1.86E+00)+	(6.14E+00)−	(5.80E−01)+	(3.43E+00)−	(1.14E+01)−	(3.92E+00)−	(3.09E+00)−	(9.87E−01)
F21	4.73E+02	4.82E+02	2.68E+02	5.64E+02	1.38E+03	3.95E+02	4.86E+02	5.27E+02	4.39E+02	5.33E+02
	(1.46E+02)$\approx$	(1.27E+02)+	(1.10E+02)+	(2.07E+02)$\approx$	(5.51E+02)−	(9.18E+01)+	(1.58E+02)+	(1.64E+02)$\approx$	(1.29E+02)$\approx$	(7.93E+02)
F22	1.20E+02	1.51E+02	1.51E+02	1.98E+02	1.61E+02	2.13E+02	4.02E+02	1.29E+02	2.40E+02	1.58E+02
	(7.82E+01)+	(8.33E+01))$\approx$	(9.70E+01)$\approx$	(1.02E+02)−	(5.62E+01)$\approx$	(7.68E+01)−	(2.49E+02)−	(6.42E+01)+	(7.78E+01)−	(6.35E+01)
F23	3.44E+02	2.00E+02	3.44E+02	3.44E+02	3.44E+02	3.44E+02	3.44E+02	3.44E+02	3.44E+02	3.44E+02
	(2.78E−13)$\approx$	(0.00E+00)+	(3.10E−13)$\approx$	(1.93E−13)$\approx$	(2.19E−05)$\approx$	(1.88E−13)$\approx$	(2.89E−13)$\approx$	(2.70E−13)$\approx$	(2.63E−13)$\approx$	(6.48E−10)
F24	2.75E+02	2.00E+02	2.71E+02	2.75E+02	2.69E+02	2.74E+02	2.71E+02	2.75E+02	2.73E+02	2.25E+02
	(1.02E+00)−	(1.15E−13)+	(2.32E+00)−	(1.04E+00)−	(1.72E+00)−	(1.17E+00)−	(2.56E+00)−	(1.38E+00)−	(1.32E+00)−	(3.58E+00)
F25	2.05E+02	2.00E+02	2.05E+02	2.06E+02	2.16E+02	2.05E+02	2.06E+02	2.06E+02	2.05E+02	2.00E+02
	(3.51E−01)−	(0.00E+00))$\approx$	(1.55E−01)−	(4.46E−01)−	(3.23E+00)−	(2.85E−01)−	(5.87E−01)−	(4.24E−01)−	(3.10E−01)−	(0.00E+00)
F26	1.00E+02	1.00E+02	1.00E+02	1.17E+02	1.18E+02	1.04E+02	1.00E+00	1.03E+02	1.10E+02	1.11E+02
	(2.04E−02)+	(3.59E−02)+	(2.89E−02)+	(3.78E+01)$\approx$	(3.26E+01)$\approx$	(1.82E+01)$\approx$	2(8.26E−02)+	(1.82E+01)+	(3.05E+01)$\approx$	(4.22E+01)
F27	3.40E+02	2.00E+02	3.12E+02	3.27E+02	3.00E+02	3.10E+02	3.84E+02	3.14E+02	3.08E+02	3.000E+02
	(3.48E+01)−	(0.00E+00)+	(1.98E+01)−	(3.10E+01)−	(2.17E−03)$\approx$	(1.93E+01)−	(5.30E+01)−	(2.36E+01)−	(1.80E+01)−	(8.45E−02)
F28	1.11E+03	2.00E+02	1.09E+03	1.27E+03	1.23E+03	1.23E+03	1.14E+03	1.28E+03	1.22E+03	1.26E+03
	(2.79E+01)+	(0.00E+00)+	(4.08E+01)+	(7.80E+01)$\approx$	(7.57E+01)$\approx$	(6.90E+01)+	(4.27E+01)+	(8.41E+01)$\approx$	(6.46E+01)+	(5.46E+01)
F29	7.98E+02	7.98E+02	8.05E+02	6.05E+02	4.89E+02	5.89E+02	7.63E+02	6.21E+02	6.22E+02	6.18E+02
	(3.57E+01)−	(2.95E+01)−	(4.53E+01)−	(1.30E+02)$\approx$	(3.02E+01)+	(1.37E+02)$\approx$	(6.12E+01)−	(1.43E+02)$\approx$	(1.06E+02)$\approx$	(4.64E+01)
F30	8.80E+03	8.84E+03	8.28E+03	9.66E+03	8.77E+03	9.37E+03	9.00E+03	9.54E+03	9.10E+03	8.48E+03
	(4.10E+02)$\approx$	(5.28E+02)−	(3.02E+02)+	(6.80E+02)−	(3.68E+02)−	(6.44E+02)−	(5.67E+02)−	(6.48E+02)−	(4.73E+02)$\approx$	(6.85E+02)
−	16	13	15	17	11	16	22	16	15	
+	8	10	11	6	5	9	5	7	7	
$\approx$	6	7	4	7	14	5	3	7	8	

“$+$” or “−” denotes that the current result is significantly better or statistical worse than the result of our IR-CMA-ES in terms of Wilcoxon’s rank sum test at a 0.05 significance level, respectively. Meanwhile, “$\approx$” represents that there is no significant difference.

**Table 4:** Results of the ten algorithms for the CEC 2014 functions with 100 in dimensionality.

Function	Average (standard deviation)									
	L-SHADE	UMOEAs-II	jSO	L-PalmDE	HS-ES	HARDDE	NDE	PaDE	CSDE	IR-CMA-ES
F1	1.56E+05	3.98E−03	1.37E+05	1.46E+05	1.20E−01	1.13E+05	9.69E+05	1.25E+05	1.09E+05	1.67E−01
	(4.43E+04)−	(5.43E−04)+	(4.54E+04)−	(4.99E+04)−	(1.77E−01)$\approx$	(3.76E+04)−	(2.99E+05)−	(4.54E+04)−	(4.80E+04)−	(3.08E−01)
F2	3.00E−13	1.83E−09	0.00E+00	1.71E−13	1.99E−09	5.31E−12	1.90E+03	2.07E−13	2.76E−13	2.13E−09
	(8.06E−14)+	(3.21E−09)$\approx$	(0.00E+00)+	(5.85E−14)$\approx$	(3.11E−09)$\approx$	(9.40E−12)$\approx$	(2.77E+03)−	(8.45E−14)$\approx$	(2.69E−13)$\approx$	(2.58E−09)
F3	4.95E−13	8.52E−09	0.00E+00	9.66E−05	1.20E−08	2.75E−12	1.10E+01	5.72E−13	1.01E−12	9.15E−09
	(1.76E−13)+	(1.29E−08)$\approx$	(0.00E+00)+	(5.29E−04)−	(2.34E−08)$\approx$	(1.85E−12)$\approx$	(1.06E+01)−	(2.37E−13)$\approx$	(8.65E−13)$\approx$	(3.57E−08)
F4	1.65E+02	1.64E+02	1.56E+02	1.29E+02	5.39E+01	1.37E+02	1.73E+02	1.29E+02	1.48E+02	5.18E+01
	(2.94E+01)−	(3.07E+01)−	(2.80E+01)−	(5.09E+01)−	(6.84+01)$\approx$	(3.45E+01)−	(3.29E+01)−	(3.32E+01)−	(1.70E+01)−	(6.35E+01)
F5	2.06E+01	2.00E+01	2.08E+01	2.04E+01	2.00E+01	2.05E+01	2.05E+01	2.05E+01	2.04E+01	2.00E+01
	(2.78E−02)−	(1.07E−04)$\approx$	(3.15E−01)−	(2.57E−01)−	(1.04E−04)$\approx$	(1.01E−01)−	(2.66E−01)−	(1.53E−01)−	(3.58E−02)−	(1.39E−04)
F6	8.82E+00	9.06E+00	4.01E+00	1.41E+01	1.64E+00	4.34E+00	5.79E+01	1.05E+01	5.71E+00	3.06E+00
	(2.14E+00)−	(2.67E+00)−	(1.76E+00)$\approx$	(1.21E+01)−	(9.16E−01)+	(1.63E+00)$\approx$	(1.60E+01)−	(2.96E+00)−	(2.54E+00)$\approx$	(1.32+00)
F7	1.89E−13	3.07E−13	0.00E+00	3.29E−04	9.74E−09	1.86E−13	2.87E−03	2.20E−13	1.21E−13	7.13E−12
	(5.45E−14)$\approx$	(9.51E−14)$\approx$	(0.00E+00)+	(1.80E−03)−	(3.14E−08)−	(5.57E−14)$\approx$	(6.72E−03)−	(4.15E−14)$\approx$	(2.88E−14)$\approx$	(1.65E−11)
F8	1.40E−03	6.12E−12	4.20E−03	1.13E−11	3.88E+00	1.43E−08	2.87E+01	1.75E−08	1.59E−07	1.15E+00
	(8.40E−04)+	(2.18E−12)+	(2.53E−03)+	(2.38E−11)+	(1.15E+00)−	(9.08E−09)+	(2.51E+01)−	(2.04E−08)+	(1.11E−07)+	(2.24E+00)
F9	3.96E+01	2.56E+01	4.45E+01	5.61E+01	2.65E+00	7.02E+01	9.87E+01	4.52E+01	5.56E+01	6.96E+00
	(5.76E+00)−	(4.92E+00)−	(6.75E+00)−	(7.50E+00)−	(1.66E+00)+	(6.32E+00)−	(3.00E+01)−	(5.10E+00)−	(5.66E+00)−	(3.52E+00)
F10	2.01E+01	8.53E+01	8.01E+01	1.85E+00	6.19E+02	4.23E−01	3.93E+01	4.24E−01	7.07E−01	2.25E+02
	(5.14E+00)+	(1.36E+02)+	(2.59E+01)+	(1.06E+00)+	(3.22E+02)−	(1.95E−01)+	(2.29E+01)+	(2.64E−01)+	(2.42E−01)+	(3.38E+02)
F11	1.08E+04	1.16E+04	1.01E+04	9.90E+03	2.07E+03	1.04E+04	1.22E+04	9.99E+03	9.90E+03	1.36E+04
	(5.16E+02)+	(8.62E+02)+	(7.02E+02)+	(7.10E+02)+	(3.66E+02)+	(4.65E+02)+	(1.33E+03)+	(4.24E+02)+	(4.39E+02)+	(1.25E+03)
F12	4.25E−01	2.52E−01	4.60E−01	4.31E−01	1.66E−02	3.95E−01	2.88E−01	3.79E−01	3.16E−01	1.37E−02
	(3.25E−02)−	(1.14E−01)−	(8.64E−02)−	(1.25E−01)−	(1.55E−02)$\approx$	(3.75E−02)−	(2.02E−01)−	(5.75E−02)−	(2.09Ee-02)−	(1.36E−02)
F13	2.40E−01	1.96E−01	3.09E−01	2.51E−01	8.78E−02	2.98E−01	2.86E−01	2.66E−01	2.65E−01	5.42E−02
	(1.36E−02)−	(4.38E−02)−	(4.29E−02)−	(3.02E−02)−	(1.55E−02)−	(2.35E−02)−	(5.84E−02)−	(1.75E−02)−	(1.78E−02)−	(3.67E−02)
F14	2.24E−01	3.25E−01	2.10E+02	2.25E−01	3.71E−01	3.14E−01	2.32E−01	3.25E−01	3.18E−01	3.23E−01
	(1.09E−02)+	(1.77E−02)$\approx$	(3.97E+01)−	(1.78E−02)+	(3.03E−02)−	(1.82E−02)$\approx$	(2.34E−02)$\approx$	(2.03E−02)$\approx$	(1.65E−02)$\approx$	(3.64E−02)
F15	1.63E+01	1.26E+01	1.55E+01	1.14E+01	1.19E+01	1.61E+01	1.19E+01	1.48E+01	1.56E+01	1.24E+01
	(1.47E+00)−	(1.14E+00)$\approx$	(1.93E+00)$\approx$	(1.75E+00)$\approx$	(1.81E+00)$\approx$	(1.43E+00)−	(1.51E+00)$\approx$	(1.31E+00)−	(8.10E−01)−	(3.69E+01)
F16	3.92E+01	4.17E+01	3.89E+01	3.93E+01	4.12E+01	3.91E+01	4.25E+01	3.88E+01	3.93E+01	4.18E+01
	(4.94E−01)$\approx$	(1.19E+00)$\approx$	(7.06E−01)$\approx$	(1.03E+00)$\approx$	(1.13E+00)$\approx$	(5.07E−01)$\approx$	(1.18E+00)−	(8.13E−01)$\approx$	(3.99E−01)$\approx$	(4.35E+00)
F17	4.02E+03	4.31E+03	3.89E+01	4.49E+03	1.53E+03	4.22E+03	9.25E+03	4.48E+03	4.32E+03	1.87E+03
	(6.58E+02)−	(6.26E+02)−	(7.05E−01)+	(7.02E+02)−	(2.16E+03)$\approx$	(6.95E+02)−	(4.15E+03)−	(6.39E+02)−	(7.27E+02)−	(2.84E+03)
F18	2.18E+02	2.23E+02	2.17E+02	2.20E+02	1.66E+00	2.24E+02	2.53E+02	2.22E+02	2.23E+02	1.46E+00
	(1.39E+01)−	(1.08E+01)−	(2.04E+01)−	(1.40E+01)−	(9.67E−01)−	(1.84E+01)−	(2.94E+01)−	(1.49E+01)−	(1.59E+01)−	(7.71E−01)
F19	9.66E+01	9.67E+01	9.11E+01	1.05E+02	7.13E+01	9.83E+01	9.16E+01	1.07E+02	9.81E+01	7.24E+01
	(2.18E+00)−	(2.35E+00)−	(1.19E+00)−	(8.80E+00)−	(2.29E+01)$\approx$	(4.98E+00)−	(1.57E+00)−	(5.40E+00)−	(2.41E+00)−	(4.98E+01)
F20	1.28E+02	1.39E+02	4.87E+01	2.54E+02	3.58E+02	9.23E+01	3.38E+02	2.19E+02	1.06E+02	3.82E+02
	(3.80E+01)+	(4.73E+01)+	(1.14E+01)+	(4.30E+01)$\approx$	(2.10E+02)$\approx$	(2.49E+01)+	(9.33E+01)$\approx$	(6.60E+01)−	(2.55E+01)+	(3.47E+02)
F21	2.14E+03	1.90E+03	9.04E+02	2.42E+03	3.20E+03	1.59E+03	1.72E+03	2.47E+03	1.78E+03	1.48E+03
	(5.44E+02)−	(5.52E+02)−	(3.73E+02)+	(4.86E+02)−	(6.71E+02)−	(4.70E+02)$\approx$	(4.51E+02)−	(5.50E+02)−	(4.81E+02)$\approx$	(7.85E+02)
F22	1.05E+03	1.05E+03	1.05E+03	1.03E+03	6.49E+02	1.11E+03	1.51E+03	9.98E+02	1.10E+03	1.00E+03
	(1.80E+02)$\approx$	(2.90E+02)$\approx$	(2.43E+02)$\approx$	(1.52E+02)$\approx$	(4.00E+02)+	(1.46E+02)−	(4.53E+02)−	(1.60E+02)$\approx$	(2.01E+02)$\approx$	(3.28E+02)
F23	3.48E+02	2.00E+02	3.48E+02	3.48E+02	3.48E+02	3.48E+02	3.48E+02	3.48E+02	3.48E+02	3.48E+02
	(1.89E−13)$\approx$	(0.00E+00)+	(0.00E+00)$\approx$	(4.16E−13)$\approx$	(3.83E−03)$\approx$	(3.96E−13)$\approx$	(3.84E−12)$\approx$	(4.32E−13)$\approx$	(3.43E−13)$\approx$	(7.97E−08)
F24	3.94E+02	2.00E+02	3.84E+02	3.90E+02	3.75E+02	3.89E+02	3.79E+02	3.91E+02	3.87E+02	2.00E+02
	(2.77E+00)−	(1.06E−11)$\approx$	(2.66E+00)−	(2.31E+00)−	(2.72E+00)−	(2.82E+00)−	(2.79E+00)−	(2.29E+00)−	(1.88E+00)−	(3.44E−10)
F25	2.00E+02	2.00E+02	2.02E+02	2.00E+02	2.01E+02	2.00E+02	2.26E+02	2.00E+02	2.00E+02	2.00E+02
	(2.08E−13)$\approx$	(0.00E+00)$\approx$	(4.79E+00)−	(3.24E−13)$\approx$	(2.90E+00)−	(2.72E−13)$\approx$	(1.04E+01)−	(3.43E−13)$\approx$	(8.44E−14)$\approx$	(2.72E+00)
F26	2.00E+02	2.00E+02	2.00E+02	2.00E+02	2.00E+02	2.00E+02	1.57E+02	2.00E+02	2.00E+02	2.00E+02
	(2.23E−13)$\approx$	(0.00E+00)$\approx$	(1.99E−13)$\approx$	(2.42E−13)$\approx$	(3.86E−02)$\approx$	(3.88E−13)$\approx$	(5.03E+01)+	(2.10E−13)$\approx$	(2.80E−13)$\approx$	(0.00E+00)
F27	3.79E+02	2.00E+02	3.43E+02	4.80E+02	3.00E+02	3.66E+02	6.71E+02	4.47E+02	3.46E+02	3.22E+02
	(4.03E+01)−	(0.00E+00)+	(3.14E+01)−	(4.88E+01)−	(1.86E−05)+	(4.28E+01)−	(7.57E+01)−	(3.76E+01)−	(2.92E+01)−	(6.28E+00)
F28	2.24E+03	2.00E+02	2.14E+03	2.45E+03	2.35E+03	2.45E+03	2.27E+03	2.63E+03	2.59E+03	2.14E+03
	(7.26E+01)−	(0.00E+00)+	(7.33E+01)$\approx$	(5.28E+02)−	(2.70E+02)−	(4.60E+02)−	(5.62E+01)$\approx$	(4.87E+02)−	(4.35E+02)−	(1.27E+02)
F29	7.59E+02	8.70E+02	7.36E+02	8.19E+02	7.30E+02	7.58E+02	1.10E+03	7.97E+02	7.94E+02	1.03E+03
	(3.67E+01)+	(1.09E+02)+	(3.06E+01)+	(8.52E+01)+	(5.88E+01)+	(3.58E+01)+	(1.86E+02)$\approx$	(6.94E+01)+	(6.84E+01)+	(2.95E+02)
F30	8.04E+03	7.93E+03	4.73E+03	6.95E+03	6.14E+03	5.44E+03	3.80E+03	6.80E+03	6.65E+03	3.27E+03
	(8.80E+02)−	(9.34E+02)−	(1.01E+03)$\approx$	(1.11E+03)−	(1.56E+03)−	(7.57E+02)−	(7.95E+02)$\approx$	(1.03E+03)−	(8.44E+02)−	(4.67E+03)
−	16	10	12	17	11	15	20	17	14	
+	8	9	10	5	6	5	3	4	5	
$\approx$	6	11	8	8	13	10	7	9	11	

“$+$” or “−” denotes that the current result is significantly better or statistical worse than the result of our IR-CMA-ES in terms of Wilcoxon’s rank sum test at a 0.05 significance level, respectively. Meanwhile, “$\approx$” represents that there is no significant difference.

It can be seen from Table 2 that, for functions with 30 dimensions, our algorithm performs significant better than L-SHADE, UMOEAs-II, jSO, L-PalmDE, HS-ES, HARDDE, NDE, PaDE and CSDE in 12, 10, 10, 13, 13, 13, 19, 11, and 12 cases. Meanwhile, our IR-CMA-ES statistically loses to the peers in 8, 10, 8, 6, 5, 8, 2, 8, and 8 cases. There is no significant difference in other cases. In short, our algorithm shows better performance than L-SHADE, jSO, L-PalmDE, HS-ES, HARDDE, NDE, PaDE and CSDE, while is comparable to UMOEAs-II.

As shown in Table 3, for functions with 50 dimensions, our algorithm performs significant better than L-SHADE, UMOEAs-II, jSO, L-PalmDE, HS-ES, HARDDE, NDE, PaDE and CSDE in 16, 13, 15, 17, 11, 16, 22, 16, and 15 cases. Meanwhile, our IR-CMA-ES statistically loses to the peers in 8, 10, 11, 6, 5, 9, 5, 7, and 7 cases. There is no significant difference in other cases. In short, our algorithm show better performance than all the peers.

According to Table 4, for functions with 100 dimensions, our algorithm performs significant better than L-SHADE, UMOEAs-II, jSO, L-PalmDE, HS-ES, HARDDE, NDE, PaDE and CSDE in 16, 10, 12, 17, 11, 15, 20, 17, and 14 cases. Meanwhile, our IR-CMA-ES statistically loses to the peers in 8, 9, 10, 5, 6, 5, 3, 4, and 5 cases. There is no significant difference in other cases. In short, our algorithm shows better better performance than the all peers.

It can be seen from Fig 1 that, the algorithm mainly based on CMA-ES, HS-ES, stagnates much early. Nevertheless, our IR-CMA-ES shows quite different from HS-ES in convergence graph.

### Experiment based on the CEC 2017 benchmark testing suite

When $D=30$, 50, and 100, the peers and our algorithm are executed 30 times for each function, respectively. The results are given in Tables 5, 6 and 7.

**Table 5:** Results of the ten algorithms for the CEC 2017 functions with 30 in dimensionality.

Function	Average (standard deviation)									
	L-SHADE	UMOEAs-II	jSO	L-PalmDE	HS-ES	HARDDE	NDE	PaDE	CSDE	IR-CMA-ES
F1	0.00E+00	9.00E−15	0.00E+00	0.00E+00	3.29E−10	7.58E−15	0.00E+00	0.00E+00	4.74E−16	0.00E+00
	(0.00E+00)$\approx$	(6.965E−15)$\approx$	(0.00E+00)$\approx$	(0.00E+00)$\approx$	(7.74E−10)$\approx$	(7.21E−15)$\approx$	(0.00E+00)$\approx$	(0.00E+00)$\approx$	(2.59E−15)$\approx$	(0.00E+00)
F2	2.84E−15	3.51E−14	0.00E+00	6.63E−15	1.26E−07	6.07E+03	4.84E−09	9.47E−16	5.68E−15	4.66E−10
	(8.67E−15)$\approx$	(3.476E−14)$\approx$	(0.00E+00)$\approx$	(1.43E−14)$\approx$	(2.11E−07)$\approx$	(3.32E+04)−	(2.459E−08)$\approx$	(5.19E−15)$\approx$	(1.16E−14)$\approx$	(6.76E−09)
F3	9.47E−15	4.93E−14	0.00E+00	1.52E−14	3.65E−10	1.71E−14	6.18E−04	7.58E−15	0.00E+00	6.78E−11
	(2.15E−14)$\approx$	(2.468E−14)$\approx$	(0.00E+00)$\approx$	(2.56E−14)$\approx$	(6.24E−10)$\approx$	(2.65E−14)$\approx$	(3.387E−03)−	(1.97E−14)$\approx$	(0.00E+00)$\approx$	(8.65E−10)
F4	5.86E+01	5.66E+01	5.86E+01	5.34E+01	3.06E+00	5.70E+01	5.91E+01	5.29E+01	5.68E+01	3.74E+01
	(2.99E−14)−	(1.07E+01)−	(2.08E−14)−	(1.77E+01)−	(1.72E+00)$+$	(1.09E+01)−	(1.695E+00)−	(1.80E+01)−	(1.08E+01)$\approx$	(4.63E+01)
F5	6.24E+00	1.23E+00	8.79E+00	1.13E+01	8.56E+00	1.26E+01	3.98E+01	8.12E+00	1.10E+01	7.63E+00
	(1.58E+00)$\approx$	(1.13E+00)$+$	(1.56E+00)$\approx$	(2.32E+00)−	(3.11E+00)$\approx$	(1.84E+00)−	(1.98E+01)−	(1.85E+00)$\approx$	(1.59E+00)−	(6.64E+00)
F6	1.14E−13	2.74E−08	1.14E−08	1.14E−13	1.14E−13	2.06E−08	4.56E−09	9.14E−09	4.56E−09	1.14E−13
	(0.00E+00)$\approx$	(6.75E−08)−	(3.46E−08)−	(2.99E−14)$\approx$	(0.00E+00)$\approx$	(4.72E−08)−	(2.50E−08)−	(3.48E−08)−	(2.50E−08)−	(0.00E+00)
F7	3.75E+01	3.30E+01	3.92E+01	4.15E+01	4.06E+01	4.29E+01	5.97E+01	3.87E+01	4.00E+01	3.83E+01
	(9.84E−01)$\approx$	(6.06E−01)$+$	(1.70E+00)−	(3.45E+00)−	(4.12E+00)−	(2.33E+00)−	(1.03E+01)−	(2.11E+00)$\approx$	(1.62e+00)$\approx$	(2.85E+00)
F8	6.70E+00	1.19E+00	9.33E+00	1.25E+01	7.79E+00	1.30E+01	5.25E+01	9.48E+00	1.13E+01	3.57E+00
	(1.55E+00)−	(1.26E+00)$+$	(1.72E+00)−	(2.96E+00)−	(2.86E+00)−	(2.61E+00)−	(1.91E+01)−	(1.46E+00)−	(1.54E+00)−	(5.80E+00)
F9	0.00E+00	0.00E+00	0.00E+00	0.00E+00	0.00E+00	0.00E+00	0.00E+00	0.00E+00	0.00E+00	0.00E+00
	(0.00E+00)$\approx$	(0.00E+00)$\approx$	(0.00E+00)$\approx$	(0.00E+00)$\approx$	(0.00E+00)$\approx$	(0.00E+00)$\approx$	(0.00E+00)$\approx$	(0.00E+00)$\approx$	(0.00E+00)$\approx$	(0.00E+00)
F10	1.49E+03	1.47E+03	1.51E+03	1.68E+03	1.04E+03	1.55E+03	2.55E+03	1.52E+03	1.54E+03	1.35E+03
	(1.91E+02)$\approx$	(2.63E+02)$\approx$	(2.21E+02)−	(3.42E+02)−	(4.12E+02)$+$	(1.90E+02)$\approx$	(6.49E+02)−	(1.74E+02)$\approx$	(2.15E+02)$\approx$	(3.72E+02)
F11	1.92E+01	2.74E+01	7.01E+00	1.13E+01	8.12E+00	5.04E+00	1.35E+01	1.81E+01	1.26E+01	1.48E+01
	(2.54E+01)$\approx$	(2.88E+01)−	(1.48E+01)$+$	(1.75E+01)$+$	(1.54E+01)$+$	(2.06E+00)$+$	(1.44E+01)$\approx$	(2.30E+01)$\approx$	(2.05E+01)$\approx$	(1.96E+01)
F12	1.24E+03	8.34E+02	1.92E+02	1.08E+03	1.28E+01	6.37E+02	4.92E+02	1.08E+03	9.23E+02	9.84E+00
	(4.08E+02)−	(3.66E+02)−	(1.03E+02)−	(4.03E+02)−	(5.43E+01)−	(3.07E+02)−	(2.28E+02)−	(3.21E+02)−	(3.68E+02)−	(4.89E+01)
F13	1.71E+01	1.56E+01	1.51E+01	1.50E+01	2.99E+01	1.63E+01	1.63E+01	1.57E+01	1.32E+01	2.86E+01
	(5.07E+00)$+$	(7.03E+00)$+$	(5.99E+00)$+$	(6.18E+00)$+$	(1.67E+01)$\approx$	(5.92E+00)$+$	(8.07E+00)$+$	(5.86E+00)$+$	(6.37E+00)$+$	(2.29E+01)
F14	2.23E+01	2.26E+01	2.23E+01	1.76E+01	1.14E+01	2.30E+01	2.05E+01	2.29E+01	2.26E+01	1.87E+01
	(1.30E+00)−	(1.43E+00)−	(9.77E−01)−	(8.33E+00)$\approx$	(1.01E+01)$+$	(2.93E+00)−	(1.00E+01)$\approx$	(1.67E+00)−	(3.76E+00)−	(2.53E+01)
F15	2.80E+00	4.04E+00	1.03E+00	4.31E+00	5.33E+00	2.17E+00	4.85E+00	2.66E+00	2.38E+00	5.14E+00
	(1.27E+00)$+$	(1.79E+00)$\approx$	(5.41E−01)$+$	(2.41E+00)$\approx$	(2.92E+00)$\approx$	(1.14E+00)$+$	(1.87E+00)$\approx$	(1.36E+00)$+$	(1.40E+00)$+$	(3.67E+00)
F16	4.64E+01	5.37E+01	6.72E+01	1.80E+02	2.64E+02	1.78E+02	2.66E+02	9.67E+01	2.06E+02	9.94E+01
	(4.88E+01)$+$	(6.39E+01)$+$	(8.71E+01)$+$	(1.35E+02)−	(2.03E+02)−	(7.25E+01)−	(2.49E+02)−	(9.06E+01)$\approx$	(9.43E+01)−	(6.64E+01)
F17	3.32E+01	4.16E+01	2.93E+01	3.27E+01	5.58E+01	3.59E+01	6.04E+01	2.94E+01	3.43E+01	5.49E+01
	(6.96E+00)$+$	(9.16E+00)$+$	(7.51E+00)$+$	(9.99E+00)$+$	(9.45E+01)$\approx$	(5.98E+00)$+$	(3.75E+01)$\approx$	(5.22E+00)$+$	(8.27E+00)$+$	(6.65E+01)
F18	2.16E+01	2.17E+01	2.01E+01	2.24E+01	1.96E+01	2.12E+01	2.31E+01	2.19E+01	2.14E+01	1.89E+01
	(1.06E+00)−	(1.09E+00)−	(3.66E+00)−	(1.71E+00)−	(5.28E+00)$\approx$	(7.21E−01)−	(5.64E+00)−	(1.62E+00)−	(8.91E−01)−	(3.78E+00)
F19	5.68E+00	7.53E+00	4.41E+00	5.41E+00	3.63E+00	6.79E+00	5.72E+00	4.84E+00	5.93E+00	4.98E+00
	(1.77E+00)$\approx$	(2.09E+00)−	(1.67E+00)$\approx$	(1.89E+00)$\approx$	(1.23E+00)$+$	(1.95E+00)−	(1.299E+00)$\approx$	(1.37E+00)$\approx$	(2.42E+00)$\approx$	(3.01E+00)
F20	3.03E+01	4.67E+01	2.89E+01	2.82E+01	1.46E+02	4.23E+01	7.59E+01	3.51E+01	4.05E+01	4.99E+01
	(5.19E+00)$+$	(1.79E+01)$\approx$	(4.49E+00)$+$	(8.35E+00)$+$	(3.13E+01)−	(1.49E+01)$\approx$	(7.73E+01)−	(6.19E+00)$+$	(9.40E+00)$\approx$	(1.56E+01)
F21	2.07E+02	1.96E+02	2.09E+02	2.11E+02	2.10E+02	2.13E+02	2.39E+02	2.08E+02	2.12E+02	2.13E+02
	(1.46E+00)$\approx$	(2.60E+01)$+$	(1.89E+00)$\approx$	(2.66E+00)$\approx$	(3.61E+00)$\approx$	(1.95E+00)$\approx$	(1.93E+01)−	(1.02E+00)$\approx$	(2.23E+00)$\approx$	(2.52E+00)
F22	1.00E+02	1.00E+02	1.00E+02	1.00E+02	1.00E+02	1.00E+02	1.00E+02	1.00E+02	1.00E+02	1.00E+02
	(0.00E+00)$\approx$	(1.15E−13)$\approx$	(0.00E+00)$\approx$	(0.00E+00)$\approx$	(2.29E−13)$\approx$	(8.30E−14)$\approx$	(0.00E+00)$\approx$	(0.00E+00)$\approx$	(0.00E+00)$\approx$	(0.00E+00)
F23	3.49E+02	3.50E+02	3.50E+02	3.49E+02	3.53E+02	3.48E+02	3.77E+02	3.45E+02	3.49E+02	3.42E+02
	(2.21E+00)−	(3.28E+00)−	(3.52E+00)−	(4.84E+00)−	(8.44E+00)−	(3.30E+00)−	(9.20E+00)−	(2.45E+00)$\approx$	(3.36E+00)−	(3.48E+00)
F24	4.26E+02	4.26E+02	4.26E+02	4.22E+02	4.19E+02	4.22E+02	4.51E+02	4.21E+02	4.22E+02	4.25E+02
	(1.87E+00)−	(1.78E+00)$\approx$	(1.75E+00)−	(2.36E+00)$+$	(5.63E+00)$+$	(3.09E+00)$+$	(1.20E+01)−	(2.20E+00)$+$	(2.78E+00)$+$	(9.50E−01)
F25	3.87E+02	3.87E+02	3.87E+02	3.87E+02	3.87E+02	3.87E+02	3.87E+02	3.87E+02	3.87E+02	3.87E+02
	(2.42E−02)$\approx$	(8.15E−01)$\approx$	(7.52E−03)$\approx$	(3.22E−02)$\approx$	(2.75E−02)$\approx$	(1.73E−02)$\approx$	(5.74E−02)$\approx$	(2.12E−02)$\approx$	(1.74E−02)$\approx$	(3.73E−02)
F26	9.23E+02	4.38E+02	9.29E+02	9.31E+02	9.06E+02	9.27E+02	1.12E+03	8.91E+02	9.24E+02	6.30E+02
	(4.03E+01)−	(2.96E+02)$+$	(3.74E+01)−	(5.15E+01)−	(1.48E+02)−	(4.80E+01)−	(3.02E+02)−	(4.18E+01)$\approx$	(3.96E+01)−	(3.85E+02)
F27	5.04E+02	5.03E+02	4.96E+02	5.07E+02	5.18E+02	4.97E+02	4.94E+02	5.06E+02	5.01E+02	5.04E+02
	(4.90E+00)$\approx$	(5.21E+00)$+$	(6.58E+00)$+$	(5.20E+00)−	(7.60E+00)−	(6.68E+00)$+$	(9.56E+00)$+$	(4.98E+00)−	(7.28E+00)$\approx$	(6.75E+00)
F28	3.40E+02	3.03E+02	3.00E+02	3.29E+02	3.24E+02	3.15E+02	3.23E+02	3.19E+02	3.21E+02	3.15E+02
	(5.36E+01)−	(1.89E+01)$+$	(2.56E−13)$+$	(4.83E+01)$\approx$	(4.44E+01)−	(3.85E+01)$\approx$	(4.740E+01)−	(4.46E+01)$\approx$	(4.35E+01)$\approx$	(3.75E+01)
F29	4.34E+02	4.35E+02	4.34E+02	4.31E+02	4.62E+02	4.37E+02	4.39E+02	4.33E+02	4.43E+02	4.59E+02
	(6.21E+00)$\approx$	(1.24E+01)$+$	(6.46E+00)$\approx$	(1.01E+01)$\approx$	(4.29E+01)$\approx$	(1.40E+01)$\approx$	(3.32E+01)$+$	(7.46E+00)$+$	(8.21E+00)$\approx$	(8.84E+01)
F30	1.99E+03	2.00E+03	1.97E+03	2.05E+03	2.06E+03	2.01E+03	2.01E+03	2.06E+03	2.00E+03	2.06E+03
	(7.32E+01)$+$	(7.07E+01)$+$	(2.40E+01)$+$	(7.01E+01)$\approx$	(4.01E+01)$\approx$	(3.76E+01)$+$	(5.90E+01)$+$	(6.21E+01)$\approx$	(3.64E+01)$+$	(3.48E+01)
−	9	8	11	11	9	13	16	7	9	
$+$	6	14	9	5	6	7	4	6	5	
$\approx$	15	8	10	14	15	10	10	17	16	

“$+$” or “−” denotes that the current result is significantly better or statistical worse than the result of our IR-CMA-ES in terms of Wilcoxon’s rank sum test at a 0.05 significance level, respectively. Meanwhile, “$\approx$” represents that there is no significant difference.

**Table 6:** Results of the ten algorithms for the CEC 2017 functions with 50 in dimensionality.

Function	Average (standard deviation)									
	L-SHADE	UMOEAs-II	jSO	L-PalmDE	HS-ES	HARDDE	NDE	PaDE	CSDE	IR-CMA-ES
F1	1.75E−14	1.30E−13	0.00E+00	1.94E−14	1.80E−09	3.55E−14	1.61E−03	2.42E−14	1.52E−14	3.59E−14
	(6.10E−15)$\approx$	(1.01E−13)$\approx$	(0.00E+00)$\approx$	(6.97E−15)$\approx$	(4.22E−09)−	(1.04E−14)$\approx$	(2.19E−03)−	(7.60E−15)$\approx$	(3.61E−15)$\approx$	(8.31E−11)
F2	4.93E−14	2.60E−11	0.00E+00	4.83E−14	4.06E−08	6.25E−10	1.47E+06	2.12E−13	6.73E−14	7.68E−11
	(3.50E−14)$\approx$	(7.94E−11)$\approx$	(0.00E+00)$\approx$	(3.74E−14)$\approx$	(6.95E−08)$\approx$	(3.27E−09)$\approx$	(6.35E+06)−	(5.47E−13)$\approx$	(1.05E−13)$\approx$	(7.34E−09)
F3	1.69E−13	2.83E−10	0.00E+00	1.63E−13	9.98E−10	2.24E−13	3.17E−01	1.53E−13	1.21E−13	3.36E−10
	(4.08E−14)$\approx$	(8.56E−10)$\approx$	(0.00E+00)$\approx$	(5.33E−14)$\approx$	(1.41E−09)$\approx$	(5.57E−14)$\approx$	(1.32E+00)−	(4.98E−14)$\approx$	(3.87E−14)$\approx$	(1.94E−10)
F4	7.13E+01	5.56E+01	4.46E+01	6.21E+01	3.18E−10	7.86E+01	5.24E+01	8.20E+01	8.03E+01	5.72E−08
	(5.36E+01)−	(4.95E+01)−	(4.17E+01)−	(4.98E+01)−	(4.29E−10)$\approx$	(4.98E+01)−	(4.35E+01)−	(4.59E+01)−	(4.42E+01)−	(3.46E−09)
F5	1.20E+01	5.13E+00	1.71E+01	2.30E+01	1.19E+00	2.65E+01	6.94E+01	1.76E+01	2.20E+01	4.16E−01
	(1.89E+00)−	(1.44E+00)−	(3.23E+00)−	(5.97E+00)−	(9.56E−01)−	(3.98E+00)−	(3.63E+01)−	(2.37E+00)−	(2.51E+00)−	(8.27E−01)
F6	4.36E−05	1.53E−06	5.96E−07	9.21E−04	6.09E−07	1.25E−07	2.25E−07	1.27E−04	2.24E−07	4.14E−07
	(2.38E−04)−	(2.43E−06)$\approx$	(5.85E−07)$\approx$	(2.18E−03)−	(6.73E−07)$\approx$	(1.28E−07)$\approx$	(7.58E−07)$\approx$	(4.83E−04)−	(3.43E−07)$\approx$	(5.82E−07)
F7	6.30E+01	5.55E+01	6.67E+01	7.00E+01	5.46E+01	7.40E+01	9.65E+01	6.47E+01	6.73E+01	5.39E+01
	(1.34E+00)−	(8.87E−01)$\approx$	(2.56E+00)−	(5.35E+00)−	(7.46E−01)$\approx$	(4.12E+00)−	(1.23E+01)−	(2.37E+00)−	(2.50E+00)−	(3.52E+00))
F8	1.18E+01	4.31E+00	1.76E+01	2.38E+01	1.49E+00	2.76E+01	5.92E+01	1.83E+01	2.32E+01	3.96E+00
	(2.65E+00)−	(1.58E+00)$\approx$	(2.80E+00)−	(5.12E+00)−	(1.22E+00)$+$	(3.06E+00)−	(2.69E+01)−	(2.16E+00)−	(2.96E+00)−	(3.38E+00)
F9	5.31E−14	2.98E−03	0.00E+00	2.98E−03	0.00E+00	4.93E−14	7.53E−02	4.17E−14	0.00E+00	0.00E+00
	(5.77E−14)$\approx$	(1.63E−02)−	(0.00E+00)$\approx$	(1.63E−02)−	(0.00E+00)$\approx$	(5.73E−14)$\approx$	(1.37E−01)−	(5.57E−14)$\approx$	(0.00E+00)$\approx$	(0.00E+00))
F10	3.08E+03	3.28E+03	3.18E+03	3.41E+03	6.56E+02	3.24E+03	4.44E+03	3.09E+03	3.25E+03	2.75E+02
	(2.76E+02)−	(5.61E+02)−	(3.73E+02)−	(4.60E+02)−	(2.65E+02)−	(2.57E+02)−	(8.90E+02)−	(3.12E+02)−	(2.37E+02)−	(1.42E+02)
F11	4.94E+01	5.07E+01	2.67E+01	7.27E+01	1.57E+01	4.35E+01	6.28E+01	6.26E+01	4.64E+01	7.68E+01
	(8.58E+00)$+$	(1.34E+01)$+$	(3.24E+00)$+$	(1.22E+01)$\approx$	(5.95E+00)$+$	(7.39E+00)$+$	(1.27E+01)$+$	(9.59E+00)$\approx$	(9.11E+00)$+$	(9.36E+00)
F12	2.26E+03	2.45E+03	1.72E+03	2.24E+03	1.71E+03	1.77E+03	2.06E+03	2.30E+03	2.30E+03	2.38E+03
	(3.94E+02)$\approx$	(5.25E+02)$\approx$	(5.30E+02)$+$	(5.49E+02)$\approx$	(3.45E+02)$+$	(4.28E+02)$+$	(4.00E+02)$+$	(5.68E+02)$\approx$	(4.95E+02)$\approx$	(3.67E+02)
F13	5.30E+01	7.47E+01	2.98E+01	5.98E+01	5.42E+01	4.87E+01	8.00E+01	6.34E+01	5.81E+01	4.89E+01
	(1.41E+01)$\approx$	(3.45E+01)−	(1.62E+01)$+$	(2.07E+01)$\approx$	(5.38E+01)$\approx$	(3.10E+01)$\approx$	(3.05E+01)−	(3.44E+01)−	(1.68E+01)$\approx$	(5.62E+01)
F14	2.91E+01	2.96E+01	2.45E+01	3.19E+01	2.05E+01	3.05E+01	3.44E+01	2.94E+01	2.90E+01	3.44E+01
	(3.63E+00)$+$	(3.14E+00)$+$	1.75E+00)$+$	(3.51E+00)$+$	(6.53E−01)$+$	(2.87E+00)$+$	(5.00E+00)$\approx$	(3.30E+00)$+$	(2.82E+00)$+$	(9.51E−01)
F15	3.82E+01	4.21E+01	2.25E+01	4.49E+01	1.57E+01	3.09E+01	4.71E+01	4.21E+01	4.24E+01	9.52E+00
	(8.53E+00)−	(1.43E+01)−	(2.21E+00)−	(1.26E+01)−	(1.40E+01)−	(6.46E+00)−	(1.85E+01)−	(1.23E+01)−	(9.27E+00)−	(5.68E+01)
F16	3.79E+02	3.16E+02	4.01E+02	3.81E+02	3.23E+02	4.83E+02	6.89E+02	3.76E+02	4.90E+02	3.06E+02
	(1.05E+02)$\approx$	(1.42E+01)$\approx$	1.41E+02)−	(1.25E+02)$\approx$	(1.27E+02)$\approx$	(9.66E+01)−	(3.02E+02)−	(1.02E+02)$\approx$	(1.50E+02)−	(5.36E+02)
F17	2.51E+02	1.47E+02	2.87E+02	3.31E+02	4.20E+02	3.41E+02	2.60E+02	2.78E+02	3.55E+02	4.17E+02
	(8.49E+01)$+$	(4.48E+01)$+$	(9.32E+01)$+$	(1.27E+02)$+$	(9.45E+01)$\approx$	(7.94E+01)$+$	(1.18E+02)$+$	(7.02E+01)$+$	(1.01E+02)$+$	(1.32E+02)
F18	3.80E+01	8.64E+01	2.42E+01	5.50E+01	2.05E+01	2.82E+01	1.56E+02	3.99E+01	3.37E+01	1.39E+01
	(9.60E+00)−	(2.59E+01)−	(1.99E+00)−	(3.10E+01)−	(2.12E−01)−	(3.35E+00)−	(4.56E+01)−	(1.25E+01)−	(6.27E+00)−	(8.93E−01)
F19	2.28E+01	2.59E+01	1.29E+01	3.60E+01	1.07E+01	2.01E+01	2.27E+01	3.04E+01	2.34E+01	3.25E+01
	(4.28E+00)$+$	(3.73E+00)$+$	(2.35E+00)$+$	(1.42E+01)$\approx$	(1.32E+00)$+$	(2.99E+00)$+$	(4.18E+00)$+$	(1.36E+01)$\approx$	(4.85E+00)$+$	(6.35E+00)
F20	1.58E+02	1.79E+02	1.00E+02	1.68E+02	5.21E+01	2.51E+02	4.89E+02	1.63E+02	2.15E+02	1.64E+02
	(6.17E+01)$\approx$	(6.67E+01)$\approx$	(4.01E+01)$+$	(1.23E+02)$\approx$	(1.23E+01)$+$	(1.03E+02)−	(1.97E+02)−	(5.57E+01)$\approx$	(7.00E+01)−	(7.82E+01)
F21	2.13E+02	1.50E+02	2.17E+02	2.26E+02	3.62E+01	2.26E+02	2.37E+02	2.18E+02	2.25E+02	2.03E+01
	(2.04E+00)−	(0.00E+00)−	(2.92E+00)−	(3.58E+00)−	(4.21E+01)$\approx$	(3.29E+00)−	(5.41E+01)−	(1.95E+00)−	(2.85E+00)−	(3.81E+01)
F22	2.34E+03	1.50E+02	1.32E+03	5.97E+02	2.26E+00	1.01E+02	3.28E+02	4.46E+02	1.16E+02	1.73E+00
	(1.73E+03)−	(0.00E+00)−	(1.74E+03)−	(1.30E+03)−	(1.17E+00)$\approx$	(3.61E+00)−	(5.78E+02)−	(1.02E+03)−	(6.27E+01)−	(2.37E+00)
F23	4.28E+02	2.51E+02	4.29E+02	4.35E+02	6.60E+02	4.31E+02	7.73E+02	4.28E+02	4.33E+02	4.95E+02
	(5.04E+00)$+$	(1.56E+00)$+$	(6.50E+00)$+$	(6.90E+00)$+$	(2.22E+01)−	(6.47E+00)$+$	(2.65E+01)−	(5.17E+00)$+$	(5.75E+00)$+$	(6.82E+00)
F24	5.06E+02	2.00E+02	5.06E+02	5.06E+02	2.00E+02	4.99E+02	3.22E+02	5.03E+02	5.04E+02	2.00E+02
	(2.57E+00)−	(0.00E+00)$\approx$	(4.05E+00)−	(5.67E+00)−	(5.29E−09)$\approx$	(4.75E+00)−	(1.23E+02)−	(7.23E+00)−	(4.46E+00)−	(1.68E−08)
F25	4.84E+02	2.00E+02	4.82E+02	4.90E+02	4.80E+02	4.89E+02	4.85E+02	4.99E+02	4.87E+02	4.16E+02
	(5.50E+00)−	(0.00E+00)$+$	(6.87E+00)−	(1.65E+01)−	(3.00E+01)−	(1.54E+01)−	(2.87E+01)−	(2.82E+01)$\approx$	(1.75E+01)−	(5.38E+01)
F26	1.14E+03	2.00E+02	1.14E+03	1.17E+03	2.00E+02	1.15E+03	3.09E+03	1.14E+03	1.18E+03	2.00E+02
	(4.68E+01)−	(0.00E+00)$\approx$	(6.53E+01)−	(6.83E+01)−	(1.16E−08)$\approx$	(6.47E+01)−	(7.73E+02)−	(7.57E+01)−	(5.12E+01)−	(2.87E−05)
F27	5.35E+02	2.00E+02	5.14E+02	5.43E+02	1.11E+03	5.25E+02	8.98E+02	5.37E+02	5.24E+02	8.50E+02
	(1.68E+01)$+$	(0.00E+00)$+$	(1.10E+01)$+$	(1.15E+01)$+$	(6.26E+01)−	(7.60E+00)$+$	(4.55E+01)−	(1.00E+01)$+$	(8.03E+00)$+$	(4.38E+01)
F28	4.78E+02	2.00E+02	4.60E+02	5.04E+02	4.73E+02	4.70E+02	4.20E+02	4.98E+02	4.87E+02	4.59E+02
	(2.43E+01)$\approx$	(0.00E+00)$+$	(8.77E+00)$\approx$	(1.24E+01)−	(4.13E+01)$\approx$	(2.10E+01)$\approx$	(4.86E+01)$+$	(1.99E+01)$\approx$	(2.46E+01)$\approx$	(6.82E+01)
F29	3.55E+02	5.04E+02	3.62E+02	3.60E+02	6.29E+02	3.76E+02	6.40E+02	3.54E+02	3.85E+02	9.02E+02
	(1.01E+01)$+$	(1.95E+02)$+$	(1.36E+01)$+$	(2.01E+01)$+$	(1.85E+02)$+$	(1.21E+01)$+$	(8.95E+01)$+$	(1.40E+01)$+$	(1.57E+01)$+$	(2.53+02)
F30	6.68E+05	5.66E+03	6.08E+05	6.26E+05	5.61E+03	6.18E+05	5.77E+03	6.20E+05	6.10E+05	5.46E+03
	(9.03E+04)−	(2.49E+02)$\approx$	(3.12E+04)−	(3.64E+04)−	(1.24E+02)$\approx$	(3.15E+04)−	(3.24E+02)$\approx$	(3.70E+04)−	(2.95E+04)−	(5.37E+02)
−	14	9	14	16	8	15	21	14	15	
$+$	7	9	10	5	7	8	6	5	7	
$\approx$	9	12	6	9	15	7	3	11	8	

“$+$” or “−” denotes that the current result is significantly better or statistical worse than the result of our IR-CMA-ES in terms of Wilcoxon’s rank sum test at a 0.05 significance level, respectively. Meanwhile, “$\approx$” represents that there is no significant difference.

**Table 7:** Results of the ten algorithms for the CEC 2017 functions with 100 in dimensionality.

Function	Average (standard deviation)									
	L-SHADE	UMOEAs-II	jSO	L-PalmDE	HS-ES	HARDDE	NDE	PaDE	CSDE	IR-CMA-ES
F1	1.56E+05	3.98E−03	1.37E+05	1.46E+05	1.20E−01	1.13E+05	9.69E+05	1.25E+05	1.09E+05	1.67E−01
	(4.43E+04)−	(5.43E−04)$+$	(4.54E+04)−	(4.99E+04)−	(1.77E−01)$\approx$	(3.76E+04)−	(2.99E+05)−	(4.54E+04)−	(4.80E+04)−	(3.08E−01)
F2	3.00E−13	1.83E−09	0.00E+00	1.71E−13	1.99E−09	5.31E−12	1.90E+03	2.07E−13	2.76E−13	2.13E−09
	(8.06E−14)$+$	(3.21E−09)$\approx$	(0.00E+00)$+$	(5.85E−14)$\approx$	(3.11E−09)$\approx$	(9.40E−12)$\approx$	(2.77E+03)−	(8.45E−14)$\approx$	(2.69E−13)$\approx$	(2.58E−09)
F3	4.95E−13	8.52E−09	0.00E+00	9.66E−05	1.20E−08	2.75E−12	1.10E+01	5.72E−13	1.01E−12	9.15E−09
	(1.76E−13)$+$	(1.29E−08)$\approx$	(0.00E+00)$+$	(5.29E−04)−	(2.34E−08)$\approx$	(1.85E−12)$\approx$	(1.06E+01)−	(2.37E−13)$\approx$	(8.65E−13)$\approx$	(3.57E−08)
F4	1.65E+02	1.64E+02	1.56E+02	1.29E+02	5.39E+01	1.37E+02	1.73E+02	1.29E+02	1.48E+02	5.18E+01
	(2.94E+01)−	(3.07E+01)−	(2.80E+01)−	(5.09E+01)−	(6.84+01)$\approx$	(3.45E+01)−	(3.29E+01)−	(3.32E+01)−	(1.70E+01)−	(6.35E+01)
F5	2.06E+01	2.00E+01	2.08E+01	2.04E+01	2.00E+01	2.05E+01	2.05E+01	2.05E+01	2.04E+01	2.00E+01
	(2.78E−02)−	(1.07E−04)$\approx$	(3.15E−01)−	(2.57E−01)−	(1.04E−04)$\approx$	(1.01E−01)−	(2.66E−01)−	(1.53E−01)−	(3.58E−02)−	(1.39E−04)
F6	8.82E+00	9.06E+00	4.01E+00	1.41E+01	1.64E+00	4.34E+00	5.79E+01	1.05E+01	5.71E+00	3.06E+00
	(2.14E+00)−	(2.67E+00)−	(1.76E+00)$\approx$	(1.21E+01)−	(9.16E−01)$+$	(1.63E+00)$\approx$	(1.60E+01)−	(2.96E+00)−	(2.54E+00)$\approx$	(1.32+00)
F7	1.89E−13	3.07E−13	0.00E+00	3.29E−04	9.74E−09	1.86E−13	2.87E−03	2.20E−13	1.21E−13	7.13E−12
	(5.45E−14)$\approx$	(9.51E−14)$\approx$	(0.00E+00)$+$	(1.80E−03)−	(3.14E−08)−	(5.57E−14)$\approx$	(6.72E−03)−	(4.15E−14)$\approx$	(2.88E−14)$\approx$	(1.65E−11)
F8	1.40E−03	6.12E−12	4.20E−03	1.13E−11	3.88E+00	1.43E−08	2.87E+01	1.75E−08	1.59E−07	1.15E+00
	(8.40E−04)$+$	(2.18E−12)$+$	(2.53E−03)$+$	(2.38E−11)$+$	(1.15E+00)−	(9.08E−09)$+$	(2.51E+01)−	(2.04E−08)$+$	(1.11E−07)$+$	(2.24E+00)
F9	3.96E+01	2.56E+01	4.45E+01	5.61E+01	2.65E+00	7.02E+01	9.87E+01	4.52E+01	5.56E+01	6.96E+00
	(5.76E+00)−	(4.92E+00)−	(6.75E+00)−	(7.50E+00)−	(1.66E+00)$+$	(6.32E+00)−	(3.00E+01)−	(5.10E+00)−	(5.66E+00)−	(3.52E+00)
F10	2.01E+01	8.53E+01	8.01E+01	1.85E+00	6.19E+02	4.23E−01	3.93E+01	4.24E−01	7.07E−01	2.25E+02
	(5.14E+00)$+$	(1.36E+02)$+$	(2.59E+01)$+$	(1.06E+00)$+$	(3.22E+02)−	(1.95E−01)$+$	(2.29E+01)$+$	(2.64E−01)$+$	(2.42E−01)$+$	(3.38E+02)
F11	1.08E+04	1.16E+04	1.01E+04	9.90E+03	2.07E+03	1.04E+04	1.22E+04	9.99E+03	9.90E+03	1.36E+04
	(5.16E+02)$+$	(8.62E+02)$+$	(7.02E+02)$+$	(7.10E+02)$+$	(3.66E+02)$+$	(4.65E+02)$+$	(1.33E+03)$+$	(4.24E+02)$+$	(4.39E+02)$+$	(1.25E+03)
F12	4.25E−01	2.52E−01	4.60E−01	4.31E−01	1.66E−02	3.95E−01	2.88E−01	3.79E−01	3.16E−01	1.37E−02
	(3.25E−02)−	(1.14E−01)−	(8.64E−02)−	(1.25E−01)−	(1.55E−02)$\approx$	(3.75E−02)−	(2.02E−01)−	(5.75E−02)−	(2.09Ee-02)−	(1.36E−02)
F13	2.40E−01	1.96E−01	3.09E−01	2.51E−01	8.78E−02	2.98E−01	2.86E−01	2.66E−01	2.65E−01	5.42E−02
	(1.36E−02)−	(4.38E−02)−	(4.29E−02)−	(3.02E−02)−	(1.55E−02)$\approx$	(2.35E−02)−	(5.84E−02)−	(1.75E−02)−	(1.78E−02)−	(3.67E−02)
F14	2.24E−01	3.25E−01	2.10E+02	2.25E−01	3.71E−01	3.14E−01	2.32E−01	3.25E−01	3.18E−01	3.23E−01
	(1.09E−02)$+$	(1.77E−02)$\approx$	(3.97E+01)−	(1.78E−02)$+$	(3.03E−02)−	(1.82E−02)$\approx$	(2.34E−02)$+$	(2.03E−02)$\approx$	(1.65E−02)$\approx$	(3.64E−02)
F15	1.63E+01	1.26E+01	1.55E+01	1.14E+01	1.19E+01	1.61E+01	1.19E+01	1.48E+01	1.56E+01	1.24E+01
	(1.47E+00)−	(1.14E+00)$\approx$	(1.93E+00)−	(1.75E+00)$\approx$	(1.81E+00)$\approx$	(1.43E+00)−	(1.51E+00)$\approx$	(1.31E+00)−	(8.10E−01)−	(3.69E+01)
F16	3.92E+01	4.17E+01	3.89E+01	3.93E+01	4.12E+01	3.91E+01	4.25E+01	3.88E+01	3.93E+01	4.18E+01
	(4.94E−01)$+$	(1.19E+00)$\approx$	(7.06E−01)$+$	(1.03E+00)$+$	(1.13E+00)$\approx$	(5.07E−01)$+$	(1.18E+00)−	(8.13E−01)$\approx$	(3.99E−01)$\approx$	(4.35E+00)
F17	4.02E+03	4.31E+03	3.89E+01	4.49E+03	1.53E+03	4.22E+03	9.25E+03	4.48E+03	4.32E+03	1.87E+03
	(6.58E+02)−	(6.26E+02)−	(7.05E−01)$+$	(7.02E+02)−	(2.16E+03)$\approx$	(6.95E+02)−	(4.15E+03)−	(6.39E+02)−	(7.27E+02)−	(2.84E+03)
F18	2.18E+02	2.23E+02	2.17E+02	2.20E+02	1.66E+00	2.24E+02	2.53E+02	2.22E+02	2.23E+02	1.46E+00
	(1.39E+01)−	(1.08E+01)−	(2.04E+01)−	(1.40E+01)−	(9.67E−01)−	(1.84E+01)−	(2.94E+01)−	(1.49E+01)−	(1.59E+01)−	(7.71E−01)
F19	9.66E+01	9.67E+01	9.11E+01	1.05E+02	7.13E+01	9.83E+01	9.16E+01	1.07E+02	9.81E+01	7.24E+01
	(2.18E+00)−	(2.35E+00)−	(1.19E+00)−	(8.80E+00)−	(2.29E+01)$\approx$	(4.98E+00)−	(1.57E+00)−	(5.40E+00)−	(2.41E+00)−	(4.98E+01)
F20	1.28E+02	1.39E+02	4.87E+01	2.54E+02	3.58E+02	9.23E+01	3.38E+02	2.19E+02	1.06E+02	3.82E+02
	(3.80E+01)$+$	(4.73E+01)$+$	(1.14E+01)$+$	(4.30E+01)$+$	(2.10E+02)$\approx$	(2.49E+01)$+$	(9.33E+01)$\approx$	(6.60E+01)$+$	(2.55E+01)$+$	(3.47E+02)
F21	2.14E+03	1.90E+03	9.04E+02	2.42E+03	3.20E+03	1.59E+03	1.72E+03	2.47E+03	1.78E+03	1.48E+03
	(5.44E+02)−	(5.52E+02)−	(3.73E+02)$+$	(4.86E+02)−	(6.71E+02)−	(4.70E+02)$\approx$	(4.51E+02)−	(5.50E+02)−	(4.81E+02)$\approx$	(7.85E+02)
F22	1.05E+03	1.05E+03	1.05E+03	1.03E+03	6.49E+02	1.11E+03	1.51E+03	9.98E+02	1.10E+03	1.00E+03
	(1.80E+02)$\approx$	(2.90E+02)$\approx$	(2.43E+02)$\approx$	(1.52E+02)$\approx$	(4.00E+02)$+$	(1.46E+02)−	(4.53E+02)−	(1.60E+02)$\approx$	(2.01E+02)$\approx$	(3.28E+02)
F23	3.48E+02	2.00E+02	3.48E+02	3.48E+02	3.48E+02	3.48E+02	3.48E+02	3.48E+02	3.48E+02	3.48E+02
	(1.89E−13)$\approx$	(0.00E+00)$+$	(0.00E+00)$\approx$	(4.16E−13)$\approx$	(3.83E−03)$\approx$	(3.96E−13)$\approx$	(3.84E−12)$\approx$	(4.32E−13)$\approx$	(3.43E−13)$\approx$	(7.97E−08)
F24	3.94E+02	2.00E+02	3.84E+02	3.90E+02	3.75E+02	3.89E+02	3.79E+02	3.91E+02	3.87E+02	2.00E+02
	(2.77E+00)−	(1.06E−11)$\approx$	(2.66E+00)−	(2.31E+00)−	(2.72E+00)−	(2.82E+00)−	(2.79E+00)−	(2.29E+00)−	(1.88E+00)−	(3.44E−10)
F25	2.00E+02	2.00E+02	2.02E+02	2.00E+02	2.01E+02	2.00E+02	2.26E+02	2.00E+02	2.00E+02	2.00E+02
	(2.08E−13)$\approx$	(0.00E+00)$\approx$	(4.79E+00)−	(3.24E−13)$\approx$	(2.90E+00)−	(2.72E−13)$\approx$	(1.04E+01)−	(3.43E−13)$\approx$	(8.44E−14)$\approx$	(2.72E+00)
F26	2.00E+02	2.00E+02	2.00E+02	2.00E+02	2.00E+02	2.00E+02	1.57E+02	2.00E+02	2.00E+02	2.00E+02
	(2.23E−13)$\approx$	(0.00E+00)$\approx$	(1.99E−13)$\approx$	(2.42E−13)$\approx$	(3.86E−02)$\approx$	(3.88E−13)$\approx$	(5.03E+01)$+$	(2.10E−13)$\approx$	(2.80E−13)$\approx$	(0.00E+00)
F27	3.79E+02	2.00E+02	3.43E+02	4.80E+02	3.00E+02	3.66E+02	6.71E+02	4.47E+02	3.46E+02	3.22E+02
	(4.03E+01)−	(0.00E+00)$+$	(3.14E+01)−	(4.88E+01)−	(1.86E−05)$+$	(4.28E+01)−	(7.57E+01)−	(3.76E+01)−	(2.92E+01)−	(6.28E+00)
F28	2.24E+03	2.00E+02	2.14E+03	2.45E+03	2.35E+03	2.45E+03	2.27E+03	2.63E+03	2.59E+03	2.14E+03
	(7.26E+01)−	(0.00E+00)$+$	(7.33E+01)$\approx$	(5.28E+02)−	(2.70E+02)−	(4.60E+02)−	(5.62E+01)−	(4.87E+02)−	(4.35E+02)−	(1.27E+02)
F29	7.59E+02	8.70E+02	7.36E+02	8.19E+02	7.30E+02	7.58E+02	1.10E+03	7.97E+02	7.94E+02	1.03E+03
	(3.67E+01)$+$	(1.09E+02)$+$	(3.06E+01)$+$	(8.52E+01)$+$	(5.88E+01)$+$	(3.58E+01)$+$	(1.86E+02)$\approx$	(6.94E+01)$+$	(6.84E+01)$+$	(2.95E+02)
F30	8.04E+03	7.93E+03	4.73E+03	6.95E+03	6.14E+03	5.44E+03	3.80E+03	6.80E+03	6.65E+03	3.27E+03
	(8.80E+02)−	(9.34E+02)−	(1.01E+03)$\approx$	(1.11E+03)−	(1.56E+03)−	(7.57E+02)−	(7.95E+02)$\approx$	(1.03E+03)−	(8.44E+02)−	(4.67E+03)
−	16	10	13	17	10	15	21	16	14	
$+$	9	9	11	7	6	6	4	5	5	
$\approx$	5	11	6	6	14	9	5	9	11	

“$+$” or “−” denotes that the current result is significantly better or statistical worse than the result of our IR-CMA-ES in terms of Wilcoxon’s rank sum test at a 0.05 significance level, respectively. Meanwhile, “$\approx$” represents that there is no significant difference.

It can be seen from Table 5 that, for functions with 30 dimensions, our algorithm performs significant better than L-SHADE, UMOEAs-II, jSO, L-PalmDE, HS-ES, HARDDE, NDE, PaDE and CSDE in 9, 8, 11, 11, 9, 13, 16, 7, and 9 cases. Meanwhile, our IR-CMA-ES statistically loses to the peers in 6, 14, 9, 5, 6, 7, 4, 6, and 5 cases. There is no significant difference in other cases. In short, our algorithm shows better performance than L-SHADE, jSO, L-PalmDE, HS-ES, HARDDE, NDE, PaDE and CSDE, while is defeated by UMOEAs-II.

As shown in Table 6, for functions with 50 dimensions, our algorithm performs significant better than L-SHADE, UMOEAs-II, jSO, L-PalmDE, HS-ES, HARDDE, NDE, PaDE and CSDE in 14, 9, 14, 16, 8, 15, 21, 14, and 15 cases. Meanwhile, our IR-CMA-ES statistically loses to the peers in 7, 9, 10, 5, 7, 8, 6, 5, and 7 cases. In short, our algorithm shows better performance than L-SHADE, jSO, L-PalmDE, HS-ES, HARDDE, NDE, PaDE and CSDE, while is comparable to UMOEAs-II.

According to Table 7, for functions with 100 dimensions, our algorithm performs significant better than L-SHADE, UMOEAs-II, jSO, L-PalmDE, HS-ES, HARDDE, NDE, PaDE and CSDE in 16, 10, 13, 17, 10, 15, 21, 16, and 14 cases. Meanwhile, our IR-CMA-ES statistically loses to the peers in 9, 9, 11, 7, 6, 6, 4, 5, and 5 cases. There is no significant difference in other cases. In short, our algorithm shows better better performance than the all peers.

### Discussion

In our IR-CMA-ES, DE with offspring-surviving selection is employed when stagnation is detected. By this means, execution may jump out of stagnation at the cost of fitness going worse. Nevertheless, population may be further optimized. Therefore, IR-CMA-ES shows better performance than the peers with CMA-ES-UMOEAs-II and HS-ES. Meanwhile, it can be seen that our algorithm performs better than the other peers.

## Conclusion

In the field of real parameter single objective optimization, algorithm based on CMA-ES, such as UMOEAs-II and HS-ES, is competitive. Nevertheless, such a type of algorithm stagnates much earlier than DE and requires to be further improved. Provided that measure to resist stagnation is applied in algorithm based on CMA-ES, better performance can be obtained. In this paper, DE is used to solve stagnation in CMA-ES. In our IR-CMA-ES, stagnation is confirmed if improving ratio of the average fitness is low for successive generations. Then, DE with offspring-surviving selection is called. If a round of DE cannot solve stagnation, one more round with more generations is executed. Our experiments are executed based on two CEC benchmark testing suites. Our algorithm is compared with five algorithms in the experiments. Experimental results show that our IR-CMA-ES performs competitively.

Our study shows that CMA-ES requires more study for real parameter single objective optimization. In the future, we will try to propose more schemes to enhance CMA-ES. Provided that stagnation in CMA-ES can be resisted better, a great progress in real parameter single objective optimization may be made.

## Methods

Population-based metaheuristics for real parameter single objective optimization, including DE and CMA-ES, tend to stagnation. Furthermore, compared with DE, CMA-ES often stagnates even earlier. In fact, the tendency of stagnation in CMA-ES can be reversed by making changes in operators. Here, we choose to resist stagnation in CMA-ES by implement DE with offspring-surviving selection because operators of DE are much simpler than those of CMA-ES for adapting. In detail, we use Eq. (1) for mutation and Eq. (3) for crossover. More importantly, offspring-surviving selection

9 $$\begin{aligned} \vec {x}_{i,g+1}=\vec {u}_{i,g} \end{aligned}$$

is employed by us. That is, offspring are always selected, while parents are certain to be eliminated from population. In this way, distribution of population varies significantly. Although fitness may go worse after the change of distribution, stagnation has been solved. Then, CMA-ES is recalled to search in a different region.

Our IR-CMA-ES is described in Algorithm 1.

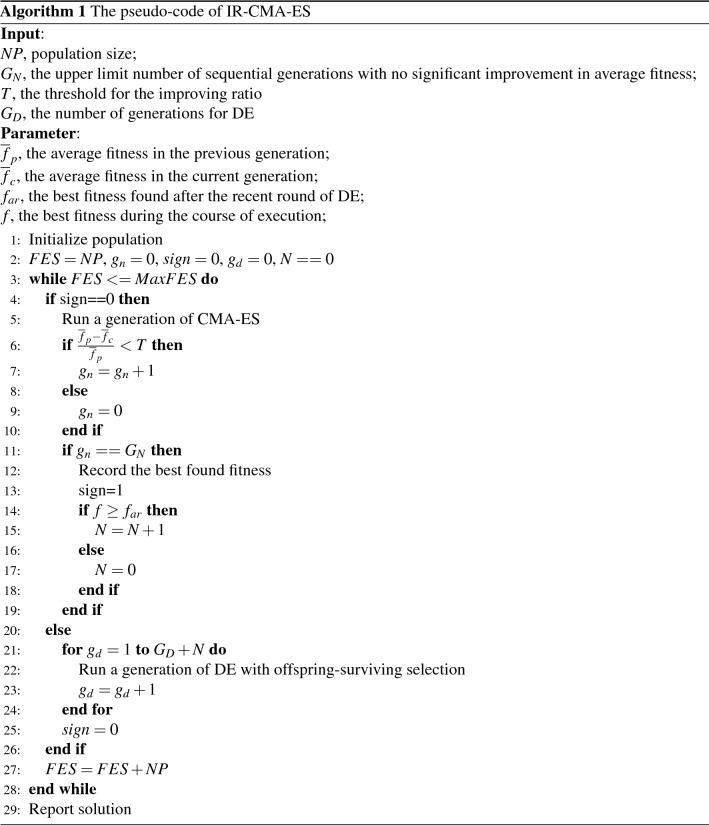



Algorithm 1 is based on the same style used in^21^. We give parameters of IR-CMA-ES as below. Firstly, parameters for CMA-ES is set according to^2^ and omitted here. Meanwhile, for DE with offspring-surviving selection, $F=1$ and $Cr=0.5$. Then, for the parameters related to our scheme, we give suggested value in Table 8.

**Table 8:** Settings for individuals redistribution based on DE.

$G_N$	500
$G_D$	1
*T*	0.001
